# Cost-effectiveness of dual maternal HIV and syphilis testing strategies in high and low HIV prevalence countries: a modelling study

**DOI:** 10.1016/S2214-109X(20)30395-8

**Published:** 2020-11-20

**Authors:** Patricia J Rodriguez, D Allen Roberts, Julianne Meisner, Monisha Sharma, Morkor Newman Owiredu, Bertha Gomez, Maeve B Mello, Alexey Bobrik, Arkadii Vodianyk, Andrew Storey, George Githuka, Thato Chidarikire, Ruanne Barnabas, Magdalena Barr-Dichiara, Muhammad S Jamil, Rachel Baggaley, Cheryl Johnson, Melanie M Taylor, Alison L Drake

**Affiliations:** a The Comparative Health Outcomes Policy & Economics Institute, University of Washington, Seattle, WA, USA; b Department of Epidemiology, University of Washington, Seattle, WA, USA; c Department of Global Health, University of Washington, Seattle, WA, USA; d Department of Medicine, University of Washington, Seattle, WA, USA; e Global HIV, Hepatitis and Sexually Transmitted Infections Programme, WHO, Geneva, Switzerland; f Pan American Health Organization and WHO, Colombia Office, Bogotá DC, Colombia; g Department of Communicable Diseases and Environmental Determinants of Health, Pan American Health Organization and WHO, Washington DC, USA; h Global Fund to Fight AIDS, Tuberculosis and Malaria, Grand-Saconnex, Switzerland; i Department of Infectious Diseases, Ukraine Country Office, WHO, Kiev, Ukraine; j Maternal and Neonatal Health, Clinton Health Access Initiative, Boston, MA, USA; k Ministry of Health, Nairobi, Kenya; l HIV Prevention Programmes, National Department of Health, Pretoria, South Africa; m Clinical Research Department, London School of Hygiene & Tropical Medicine, London, UK; n Division of Sexually Transmitted Diseases Prevention, Centers for Disease Control and Prevention, Atlanta, GA, USA

## Abstract

**Background:**

Dual HIV and syphilis testing might help to prevent mother-to-child transmission (MTCT) of HIV and syphilis through increased case detection and treatment. We aimed to model and assess the cost-effectiveness of dual testing during antenatal care in four countries with varying HIV and syphilis prevalence.

**Methods:**

In this modelling study, we developed Markov models of HIV and syphilis in pregnant women to estimate costs and infant health outcomes of maternal testing at the first antenatal care visit with individual HIV and syphilis tests (base case) and at the first antenatal care visit with a dual rapid diagnostic test (scenario one). We additionally evaluated retesting during late antenatal care and at delivery with either individual tests (scenario two) or a dual rapid diagnosis test (scenario three). We modelled four countries: South Africa, Kenya, Colombia, and Ukraine. Strategies with an incremental cost-effectiveness ratio (ICER) less than the country-specific cost-effectiveness threshold (US$500 in Kenya, $750 in South Africa, $3000 in Colombia, and $1000 in Ukraine) per disability-adjusted life-year averted were considered cost-effective.

**Findings:**

Routinely offering testing at the first antenatal care visit with a dual rapid diagnosis test was cost-saving compared with the base case in all four countries (ICER: –$26 in Kenya,–$559 in South Africa, –$844 in Colombia, and –$454 in Ukraine). Retesting during late antenatal care with a dual rapid diagnostic test (scenario three) was cost-effective compared with scenario one in all four countries (ICER: $270 in Kenya, $260 in South Africa, $2207 in Colombia, and $205 in Ukraine).

**Interpretation:**

Incorporating dual rapid diagnostic tests in antenatal care can be cost-saving across countries with varying HIV prevalence. Countries should consider incorporating dual HIV and syphilis rapid diagnostic tests as the first test in antenatal care to support efforts to eliminate MTCT of HIV and syphilis.

**Funding:**

WHO, US Agency for International Development, and the Bill & Melinda Gates Foundation.

## Introduction

Dual elimination of mother-to-child transmission (MTCT) of HIV and syphilis is a public health priority. Worldwide, 1·4 million maternal HIV infections and 988 000 maternal syphilis infections occur annually.^1^ Although maternal treatment is highly effective at preventing MTCT of both HIV and syphilis, gaps in maternal testing and treatment coverage lead to 180 000 infant HIV infections, 355 000 adverse congenital syphilis birth outcomes, and 306 000 non-clinical congenital syphilis cases every year.^1^, ^2^ WHO has set goals to reach elimination of MTCT of HIV and syphilis, including at least 95% of pregnant women receiving antenatal care, 95% tested for HIV and syphilis, and 95% treated for their infection(s).^3^, ^4^, ^5^ Yet, by 2020, only 14 countries had received validation by WHO for achieving the elimination of paediatric HIV or congenital syphilis.^1^, ^6^ Globally, as HIV testing coverage has increased, more pregnant women with HIV are aware of their status, of whom 85% have accessed treatment; whereas, only 66% of pregnant women are tested for syphilis, of whom 78% receive treatment.^1^, ^7^ Global efforts for prevention of MTCT (PMTCT) of HIV have led to substantial reductions in new paediatric HIV infections, but PMTCT of syphilis has received considerably less attention and success.^1^

Integrating syphilis testing and treatment into existing HIV PMTCT programmes might avert additional syphilis morbidity and mortality. 57% of congenital syphilis cases resulting in adverse birth outcomes were attributed to an absence of syphilis screening for women attending antenatal care.^1^ Testing coverage for HIV is often several times higher than for syphilis, suggesting that integrated testing could improve syphilis test coverage.^8^ Although rapid diagnostic tests are standard for HIV, many countries still rely on laboratory-based testing for syphilis. Three dual HIV and syphilis rapid diagnostic tests, which allow concurrent antenatal care testing that circumvents laboratory testing obstacles for syphilis, are WHO prequalified.^9^

**Research in context**

**Evidence before this study**

Globally, HIV testing coverage is high during pregnancy; by contrast, syphilis testing coverage is frequently lower, but it could be increased if combined with HIV testing. As countries strive to achieve elimination of mother-to-child transmission of both infections, studies that identify strategies to efficiently optimise implementation of maternal HIV and syphilis testing are crucial to maximise the number of infant infections prevented. We searched PubMed for studies published from the inception of the database to July 28, 2020, with no language restrictions. Using the search terms “HIV infections” OR “HIV infections/transmission” OR “HIV” OR “syphilis, congenital” AND “mass screening” OR “screening”(tiab) OR “testing”(tiab) AND “repeat”(tiab) OR “retest”(tiab) AND “maternal”(tiab) OR “pregnancy” AND “cost-effectiveness” OR “cost-benefit analysis” OR “mass screening/economics” eight studies were found with this specific combination. Previous cost-effectiveness analyses of dual HIV and syphilis testing or syphilis retesting during pregnancy have focused on a single country. Dual HIV and syphilis testing was shown to be cost-effective in Malawi and retesting for syphilis has shown mixed cost-effectiveness results in the USA. None of the previously published modelling analyses provided results relevant to a variety of contexts and settings.

**Added value of this study**

To our knowledge, this is the first study to model the health and economic effects of using a single rapid diagnostic test for both HIV and syphilis in the context of antenatal care in multiple countries that represent diverse geographical and HIV and syphilis prevalence settings. To our knowledge, this exploratory analysis of retesting for syphilis and HIV using the dual rapid diagnostic test is the first analysis of the cost-effectiveness of maternal retesting for syphilis outside the USA, and the first cost-effectiveness analysis of retesting using a dual rapid diagnostic test.

**Implications of all the available evidence**

We find that using a dual rapid diagnostic test for HIV and syphilis at the first antenatal care visit is cost-saving compared with using a rapid HIV test and laboratory-based syphilis test in all four countries evaluated, regardless of HIV prevalence. Dual rapid diagnostic test for HIV and syphilis at the first antenatal care visit is projected to avert many congenital syphilis cases and disability-adjusted life-years, but similar numbers of infant HIV infections as independent HIV and syphilis tests. Retesting in late pregnancy with a dual rapid diagnostic test was more effective and less costly than independent HIV and syphilis tests during late pregnancy. This analysis highlights potential health and economic benefits of integrating maternal HIV and syphilis testing. Programmes might consider incorporating dual rapid diagnostic tests for HIV and syphilis as a cost-effective approach within antenatal care to improve maternal and infant health outcomes.

With restricted resources (including for programmes to support funding for staff training, time to offer testing, and test kits) and a need to prioritise highly effective interventions in resource-limited settings, policy makers need health economic evidence to decide where and how to implement dual HIV and syphilis testing. Dual HIV and syphilis testing in antenatal care has been shown to be a cost-effective approach in Malawi,^9^ but benefits and costs of this approach in diverse geographical regions with different health systems and HIV and syphilis burden have not been examined.

We aimed to compare the use of a dual rapid diagnostic test with individual tests at the first antenatal care visit and assess the effect of retesting for HIV and syphilis during pregnancy using a dual rapid diagnostic test. Many countries already retest for HIV during late antenatal care and delivery,^10^ and retesting for HIV during the third trimester is recommended by WHO in high HIV-burden settings and key populations;^11^ however, some low HIV-burden settings might also consider retesting in their efforts to achieve elimination of MTCT of HIV (Meisner J and Roberts DA, unpublished). The use of retesting for syphilis during pregnancy has not previously been reported for countries with varied HIV and syphilis prevalence.^12^, ^13^

## Methods

### Model overview and testing scenarios

Using a Markov decision analytical model, we did a cost-effectiveness analysis of maternal HIV and syphilis testing using individual tests and dual rapid diagnostic tests in Kenya, South Africa, Colombia, and Ukraine—four countries that represent a range of HIV prevalence, syphilis prevalence, and geographical settings. Countries were classified as having low (<5%; Colombia and Ukraine), intermediate (5–15%; Kenya), and high (≥15%; South Africa) HIV prevalence.^14^ We compared four HIV and syphilis testing scenarios, varying the assay type and timing of testing (table 1). The primary analysis assessed the cost-effectiveness of dual HIV and syphilis testing compared with individual tests at the first antenatal care visit, and the secondary analysis evaluated the effect of retesting for HIV and syphilis during late antenatal care. We modelled the same test types across all four countries because of heterogeneity within countries and to enable comparison between countries. We modelled the use of dual rapid diagnostic test and individual HIV and syphilis tests followed by confirmatory testing. We assumed HIV diagnoses followed two consecutive reactive rapid diagnostic tests. Individual syphilis testing was modelled as a laboratory-based rapid plasma reagin test with confirmatory testing for reactive results with the *Treponema pallidum* haemagglutination assay. No confirmatory test for syphilis was modelled for dual rapid diagnostic test scenarios.

**Table 1:** Dual HIV and syphilis testing models scenarios

	**First ANC visit**		**Late ANC visit**	
	Syphilis	HIV	Syphilis	HIV
Base case	RPR and TPHA	Rapid	No test	No test
Scenario one	Dual	Dual	No test	No test
Scenario two	RPR and TPHA	Rapid	RPR and TPHA	Rapid
Scenario three	Dual	Dual	Dual	Dual

Base case used independent tests at country-specific timepoints. Scenario one used dual HIV and syphilis testing with no retesting during late ANC. Scenario two used independent HIV and syphilis tests with retesting during late ANC. Scenario three used dual HIV and syphilis testing with retesting during late ANC. Dual test is a single, point-of-care rapid test for HIV and syphilis. Independent tests include rapid HIV tests and laboratory-based syphilis tests (RPR and TPHA to confirm reactive results). Late ANC occurred from 36 to 39 weeks' gestation. ANC=antenatal care. RPR=rapid plasma reagin. TPHA=*Treponema pallidum* haemagglutination assay.

In the base case scenario, we assumed that HIV and syphilis testing with individual tests was done at the first antenatal care visit, defined by country-specific average gestational age at the first visit. In scenario one, we modelled testing at the first antenatal care visit using a dual rapid diagnostic test. Additionally, because maternal HIV retesting is already recommended in many countries, we modelled two scenarios that incorporated maternal HIV retesting to address operational considerations. To estimate the relative health and economic effect attributed to HIV versus syphilis, we compared retesting with individual tests (scenario two) with retesting during late antenatal care and delivery with a dual rapid diagnostic test (scenario three). Retesting scenarios included women who missed HIV or syphilis testing at first antenatal care visit (ie, because they did not attend antenatal care before the gestational age when retesting would occur or because of gaps in test coverage). Retesting scenarios also allowed for HIV retesting at delivery for women not tested during late antenatal care (dual and individual tests), but not for syphilis retesting at delivery. For scenarios in which dual rapid diagnostic tests were modelled (scenarios one and three), we assumed that all women tested received the test regardless of previous HIV diagnosis or syphilis infection.

### Model structure, parameterisation, and assumptions

Maternal HIV and syphilis were modelled using separate Markov models (appendix pp 1–2). The HIV model was adapted from a previous model of maternal HIV retesting, and reflects HIV prevalence, incidence, disease progression, and treatment during pregnancy and post partum using 54 Markov states (six maternal HIV states by nine antenatal and post partum periods; Meisner J and Roberts DA, unpublished). The congenital syphilis model reflects syphilis prevalence, incidence, testing, and treatment using eight maternal syphilis states with time-varying transitions. We assumed risk of MTCT of HIV and syphilis and associated infant health outcomes were independent. Risk of infant HIV was modelled for 1 year after birth. The time step in both models was 1 week, and the time horizon was 20 years after birth—a common time horizon that allows our results to be directly compared with other cost-effectiveness analyses. The model was built in Microsoft Excel (2016).

Models were parametrised using values from the literature, in-country contacts, and expert opinion; country-specific estimates were used when available (table 2). Although HIV and syphilis models were constructed separately, they share common model parameter inputs. Both models incorporate disease prevalence, incidence, antenatal care attendance, test coverage, test sensitivity and specificity, maternal and infant mortality rates, and probability of infant infection. For each country, we modelled a population of pregnant women equal to the estimated number of annual pregnancies, or the number of livebirths if the number of pregnancies was unavailable. All pregnancies were assumed to be singleton. Fetal loss because of syphilis was modelled, but fetal loss because of HIV was not.

**Table 2:** Key model parameters

			**Kenya**	**South Africa**	**Colombia**	**Ukraine**
Population of pregnant women			1 631 470	1 100 699	346 409	363 946
HIV risk						
	HIV prevalence		6·1%	3·1%	0·4%	0·7%
		Maternal HIV incidence rate (per person-week)				
		Before first ANC visit during pregnancy	0·000331	0·000227	0·00001	0·000002
		Between first ANC visit and delivery	0·000331	0·000739	0·00002	0·000004
		Delivery to 6 weeks post partum	0	0	0	0
		6 weeks to 12 months post partum	0·000269	0·0009	0·00003	0·000003
		Duration of incident maternal HIV infection (weeks)	9	9	9	9
	HIV testing and prevention					
		Test kit unavailable	5·0%	5·0%	0·0%	0·0%
		Test acceptance	84·0%	98·0%	89·0%	97·2%
		Received test results	97·8%	98·0%	100·0%	100·0%
		HIV rapid test sensitivity in early infection	66·7%	66·7%	66·7%	66·7%
		HIV rapid test sensitivity in chronic infection	100·0%	100·0%	100·0%	100·0%
		HIV rapid test specificity	98·9%	98·9%	98·9%	98·9%
		Dual test sensitivity in early infection	66·7%	66·7%	66·7%	66·7%
		Dual test sensitivity in chronic infection	100·0%	100·0%	100·0%	100·0%
		Dual test specificity	98·9%	98·9%	98·9%	98·9%
		MTCT rate per week, acute maternal infection	0·005–0·029	0·005–0·029	0·005–0·029	0·005–0·029
		MTCT rate per week, chronic maternal infection	0·0005–0·023	0·0005–0·023	0·0005–0·023	0·0005–0·023
		Maternal PrEP use	0·0%	0·0%	0·0%	0·0%
Health-care visits						
	Attended first ANC visit		96·0%	94·0%	97·4%	99·8%
	Attended late ANC visit		92·7%	78·0%	88·1%	90·0%
	Facility delivery		61·8%	96·0%	98·9%	99·0%
	Gestational age at first ANC visit (weeks)		22	18	15	10
	Gestational age at late ANC visit (weeks)		33	36	24	28
	Gestational age at delivery (weeks)		39	39	39	39
ARV coverage						
	Maternal ART use		91·0%	87·0%	87·8%	95·0%
	Virally suppressed women with HIV		88·1%	72·0%	88·4%	88·1%
	Weekly ART dropout (%)		0·3%	0·3%	0·3%	0·3%
	HIV-exposed infants receiving ARVs		93·6%	98·7%	96·0%	98·4%
	HIV-infected infants receiving ART*		61·0%	63·0%	57·9%	95·0%
Breastfeeding						
	Not breastfeeding in early post partum (0–6 weeks)		2·5%	34·0%	97·9%	95·0%
	Not breastfeeding in mid post partum (6 weeks to 6 months)		21·2%	45·0%	97·9%	99·0%
	Not breastfeeding in late post partum (6–12 months)		33·4%	63·0%	97·9%	99·0%
Maternal mortality rate (per person-week)						
	During pregnancy		0·0001	0·0001	0·00002	0·00002
	Delivery to 6 weeks post partum		0·0006	0·0002	0·0001	0·00003
	6 weeks to 12 months post partum		0·0001	0·0001	0·00002	0·00002
Neonatal and infant mortality (per person-week) and survival						
	Stillbirth, syphilis positive (mother not treated)		21·0%	21·0%	21·0%	21·0%
	Stillbirth, syphilis positive (mother treated)		3·8%	3·8%	3·8%	3·8%
	Neonatal mortality, birth to 6 weeks (mother had syphilis and not treated)		9·0%	9·0%	9·0%	9·0%
	Neonatal mortality, birth to 6 weeks (mother had syphilis and treated)		1·8%	1·8%	1·8%	1·8%
	Neonatal mortality, birth to 6 weeks (syphilis negative and HIV negative or HIV negative and on ART)		0·5%	0·3%	0·2%	0·1%
	Infant mortality, >6 weeks to 12 months		0·03%	0·03%	0·01%	0·01%
	Survival to 1 year, HIV negative or HIV positive and on ART		96·4%	96·7%	98·7%	99·2%
	Survival to 1 year, HIV positive and not on ART		64·8%	64·8%	64·8%	64·8%
Syphilis						
	Syphilis prevalence		1·2%	2·0%	0·41%	2·5%
	Maternal syphilis incidence rate (per person-week)		0·00008	0·00008	0·00004	0·000015
	Laboratory-based test sensitivity for syphilis		80·0%	80·0%	80·0%	80·0%
	Laboratory-based test specificity for syphilis		100·0%	100·0%	100·0%	100·0%
	Dual RDT sensitivity for syphilis		87·0%	87·0%	87·0%	87·0%
	Dual RDT specificity for syphilis		99·5%	99·5%	99·5%	99·5%
	Test correction factor†		52·9%	52·9%	52·9%	52·9%
	Test coverage (laboratory-based testing)‡		73·0%	83·0%	62·8%	98·0%
	Received treatment (laboratory-based testing)		50·0%	90·0%	90·8%	99·0%
	Received treatment (dual testing)		62·5%	92·5%	93·1%	99·3%
HIV costs (US$)						
	Third generation rapid screening per woman		$2·64	$7·72	$6·68	$3·99
	True-positive screening tests per woman*		$3·68	$11·39	$8·53	$4·18
	False-positive screening tests per woman*		$26·39	$34·17	$74·83	$19·80
	Maternal ART, per week per woman		$4·86	$4·79	$18·89	$32·84
	Infant ARV prophylaxis (total cost) per infant		$2·32	$3·82	$52·10	$4·00
	Maternal PrEP, per week per woman		$6·19	$6·19	$18·89	$19·38
	Infant ART, per week per infant		$6·73	$5·46	$18·89	$32·84
Syphilis costs						
	RPR test screening per woman		$3·09	$9·32	$6·92	$0·63
	TPHA test screening per woman		$0·59	$3·04	$3·43	$1·14
	Dual test screening per woman		$5·79	$8·69	$8·21	$1·99
	Benzathine benzylpenicillin injection, maternal treatment per woman		$0·64	$0·60	<$0·01	$3·00
	Intravenous benzathine benzylpenicillin, infant treatment per infant		$1·42	$1·42	$1·42	$1·42
	Paediatric inpatient, per day per infant		$8·41	$72·81	$62·87	$28·84

Details and sources provided in appendix (pp 18–30). All costs reported in 2017 US$. ANC=antenatal care. ART=antiretroviral therapy. ARV=antiretrovirals. MCH=maternal and child health. MTCT=mother-to-child transmission. PrEP=pre-exposure prophylaxis. RDT=rapid diagnostic test. RPR=rapid plasma reagin. TPHA=*Treponema pallidum* hemagglutination assay.

* Based on the percent of infants with early infant diagnosis.

† Test correction factor is the probability that a woman who tested positive for syphilis has an active syphilis infection.

‡ Inclusive of stock-outs.

### Health effect

We modelled infant HIV infection, congenital syphilis, and infant death. Infant HIV infections and deaths were estimated on the basis of total exposure to HIV in utero and post partum, with probabilities dependent on new versus established maternal HIV infection (on the basis of Fiebag stages), maternal viral suppression, maternal antiretroviral therapy (ART), infant ART, and breastfeeding practices. Congenital syphilis outcomes (stillbirth, neonatal death, preterm birth and low birthweight, clinical congenital syphilis, and non-clinical syphilis) were estimated at birth and modified by receipt and timing of adequate maternal syphilis treatment during pregnancy.^15^ We followed WHO guidelines on the definition of adequate syphilis treatment as at least 30 days before birth (modelled as ≤32 weeks) to prevent fetal infection, and set probabilities of adverse infant outcomes for later treatment equal to those for no treatment. We considered all infants born to untreated and late treated mothers to have congenital syphilis, regardless of symptoms.^6^ We accounted for duplication of outcomes between the HIV and syphilis models by calculating infant co-infection as the joint probability of syphilis and HIV infection in infants, assuming independence. Syphilis-related stillbirths and neonatal deaths were subtracted from the population at delivery in the HIV model.

We converted infant infections and adverse birth outcomes into disability-adjusted life-years (DALYs; appendix pp 16–17).^16^ Total DALYs were the sum of years lived with disability and years of life lost up to the age of 20 years. We assumed DALYs for each stillbirth and neonatal death were equal. We used disability weights from the Global Burden of Disease study and standard formulas for calculating co-infection disability weights.^16^, ^17^ For HIV-infected infants on ART at 1 year, the disability weight for HIV while on ART was applied over the 20-year time horizon. For HIV-infected infants not on ART at 1 year, we assumed 90% of time was spent with symptomatic HIV and 10% with AIDS over the 20-year time horizon. For infants with congenital syphilis, the disability weight was applied only to the first 3 years of life. DALYs were discounted at 3% annually.^18^

### Costs and cost-effectiveness

We used a health-care system perspective for the model. Costs of supplies, labour, and treatment were obtained from the literature, in-country contacts, and expert opinion (appendix pp 18–30). Empirical time-motion data collected in Kenya were used to calculate HIV testing costs in the country and estimates of time to testing were applied to other regions with country-specific labour and supply costs. Only incremental costs associated with testing and treatment were included. We excluded start-up costs (eg, training) and distribution costs. Costs given in local currency units were converted to US$ using exchange rates from June of the year they were collected. All costs were adjusted to 2017 US$ using the US gross domestic product deflator and discounted at 3% annually.^19^

The incremental cost-effectiveness ratio (ICER)—defined as the incremental costs divided by DALYs averted—was calculated by comparing scenario one (dual test at first antenatal care visit) with the base case. Retesting scenarios were compared with the next-best scenario (by DALYs). Dominated scenarios, those that were more costly and less effective than an alternative, were excluded from calculations.^20^ ICERs less than the country-specific cost-effectiveness threshold were considered cost-effective. We used the following thresholds based on estimated opportunity cost of health investment foregone: US$500 (Kenya),^21^, ^22^ $750 (South Africa),^23^ $1000 (Ukraine),^24^ and $3000 (Colombia)^25^ per DALY averted.

### Sensitivity analysis

Uncertainty analyses were done for scenario one. One-way sensitivity analyses were used to assess the effect of changing individual model parameters, including HIV and syphilis prevalence, test coverage, test characteristics, gestational age at first antenatal care visit, and costs. We used low and high parameter values from confidence interval bounds for test characteristics, and 20% relative changes for all other parameters. We also did scenario implementation analyses to estimate the effect of epidemic changes (declines in HIV and syphilis prevalence) or HIV test acceptance on cost-effectiveness, calculated using Microsoft Excel (2016).

### Role of the funding source

Collaborators from WHO were involved in study design, interpretation of results, and manuscript development; no other funders had a role in design, analysis, or interpretation. All collaborators had full access to all the data in the study. ALD had final responsibility for the decision to submit for publication, with concurrence from all authors.

## Results

We modelled populations of 1 631 470 pregnant women in Kenya, 1 100 699 in South Africa, 346 409 in Colombia, and 363 946 in Ukraine. In our models, routinely offering a dual HIV and syphilis rapid diagnostic test at the first antenatal care visit (scenario one) was more effective and less costly compared with individual HIV rapid diagnostic tests and laboratory assays for syphilis in all four countries (table 3). Per DALY averted, a dual rapid diagnostic test at the first antenatal care visit saved $26 in Kenya, $559 in South Africa, $844 in Columbia, and $545 in Ukraine. Dual rapid diagnostic tests at the first antenatal care visit averted 3005 congenital syphilis cases in Kenya, 2909 in South Africa, 403 in Colombia, and 639 in Ukraine and reduced DALYs by 9770 in Kenya, 9358 in South Africa, 1258 in Colombia, and 1980 in Ukraine. The PMTCT potential for HIV using the dual rapid diagnostic test and independent tests was similar in Ukraine and Columbia but resulted in more HIV infections in Kenya (one) and South Africa (five).

**Table 3:** Health effect and cost-effectiveness of maternal HIV and syphilis testing scenarios

	**Total costs (US$)**	**Total HIV infections**	**Total syphilis infections**	Total DALYs*	**Incremental costs (US$)**	**Incremental DALYs averted**	**ICER**†	**Comparator**
**Kenya**								
Base case	$62 924 984	13 468	18 832	1 030 415	..	..	..	..
Scenario one	$62 671 902	13 470	15 827	1 020 644	−$253 082	9770	−$26‡	Base case
Scenario two	$67 356 499	10 943	18 832	1 015 467	$4 684 597	5177	Dom	..
Scenario three	$66 707 622	10 944	15 827	1 005 693	$4 035 720	14 951	$270§	Scenario one
**South Africa**								
Base case	$151 601 450	27 012	12 257	745 434	..	..	..	..
Scenario one	$146 367 385	27 017	9348	736 077	−$5 234 065	9358	−$559‡	Base case
Scenario two	$162 156 341	23 829	12 257	727 129	$15 788 956	8947	Dom	..
Scenario three	$151 135 725	23 834	9348	717 766	$4 768 340	18 311	$260§	Scenario one
**Colombia**								
Base case	$5 855 930	145	1281	73 133	..	..	..	..
Scenario one	$4 794 810	145	878	71 875	−$1 061 120	1258	−$844‡	Base case
Scenario two	$9 279 059	122	916	71 842	$4 484 248	33	Dom	..
Scenario three	$7 295 786	122	564	70 742	$2 500 976	1133	$2207§	Scenario one
**Ukraine**								
Base case	$8 801 528	71	2333	54 818	..	..	..	..
Scenario one	$7 902 302	71	1694	52 837	−$899 226	1980	−$454‡	Base case
Scenario two	$10 327 010	67	890	50 432	$2 424 707	2405	Dom	..
Scenario three	$8 588 217	67	593	49 486	$685 914	3352	$205§	Scenario one

Base case used independent tests specific to each setting. Scenario one used dual HIV and syphilis testing with no retesting during late ANC. Scenario two used independent HIV and syphilis tests with retesting during late ANC. Scenario three used dual HIV and syphilis testing with retesting during late ANC. ANC=antenatal care. DALY=disability-adjusted life-year. ICER=incremental cost-effectiveness ratio. Dom=dominated (more costly and less effective than dominating scenario).

* DALYs averted and incremental costs are compared with the next most effective (by DALYs) scenario. Individual tests are HIV rapid tests and rapid plasma regain tests for syphilis; *Treponema pallidum* hemagglutination assay used for confirmatory syphilis testing for reactive rapid plasma regain results. Reactive rapid HIV tests and dual HIV tests confirmed with an additional HIV rapid test.

† Calculated as incremental costs (in 2017 US$) divided by DALYs averted compared with the next most effective (by DALYs) scenario, with dominated scenarios removed.

‡ Indicates scenario is cost-saving.

§ Indicates scenario is cost-effective compared to country-specific cost-effectiveness threshold.

Retesting in late pregnancy with a dual rapid diagnostic test (scenario three) was more effective and less costly than retesting in late pregnancy with individual tests (scenario two). Retesting with a dual rapid diagnostic test averted 2526 additional HIV infections in Kenya and 3184 in South Africa compared with testing with the rapid diagnostic test only at the first antenatal care visit (scenario one). Retesting with the dual rapid diagnostic test was cost-effective in all countries (ICER in Kenya $270, South Africa $260, Colombia $2207, and Ukraine $205). However, in countries with low HIV prevalence, fewer additional HIV cases were averted by retesting in Colombia (23) and Ukraine (four). When retesting occurred by 32 weeks, 352 additional congenital syphilis cases were averted in Colombia and 297 were averted in Ukraine. By contrast, retesting did not avert any additional congenital syphilis cases in Kenya or South Africa because testing was done after 32 weeks.

**Figure 1:**
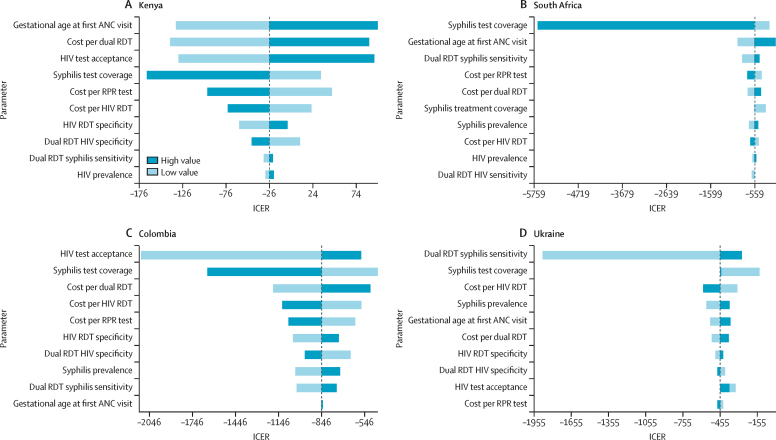
One-way sensitivity analysis for scenario one in Kenya (A), South Africa (B), Columbia (C), and Ukraine (D) Scenario one used dual HIV and syphilis testing with no retesting during late ANC. One-way sensitivity plots, comparing scenario one with the base case under high and low alternative parameter values. High and low bound represent CI bounds for available test characteristics, and 20% relative changes for all other modelled parameter values. For each country the plot contains the ten parameters to which the ICER is most sensitive. ICER was measured as US$ per DALY averted. ANC=antenatal care. DALY=disability-adjusted life-years. ICER=incremental cost-effectiveness ratio. RDT=rapid diagnostic test. RPR=rapid plasma regain.

The findings from scenario one were most sensitive to gestational age at first antenatal care visit, syphilis test coverage, overall cost of testing with dual rapid diagnostic tests, and HIV test acceptance, but were robust to parameter uncertainty (figure 1). ICER outcomes in South Africa were particularly sensitive to syphilis testing coverage, which varied between 66% and 100%; by contrast, we varied coverage in Kenya between 58% and 88%. Although ICERs were below the cost-effectiveness threshold in all one-way sensitivity analyses, ICER values for Kenya moved from cost-saving to cost-effective when gestational age at the first antenatal care visit, overall testing cost for the dual rapid diagnostic test, HIV test acceptance, syphilis testing coverage, and overall testing cost per rapid plasma regain were modified. In Colombia, ICERs were most sensitive to HIV test acceptance, syphilis testing coverage, and overall testing costs for the dual rapid diagnostic test, HIV rapid diagnostic test, and rapid plasma reagin tests. In Ukraine, ICERs were most sensitive to changes to dual rapid diagnostic test sensitivity for syphilis and syphilis testing coverage.

**Figure 2:**
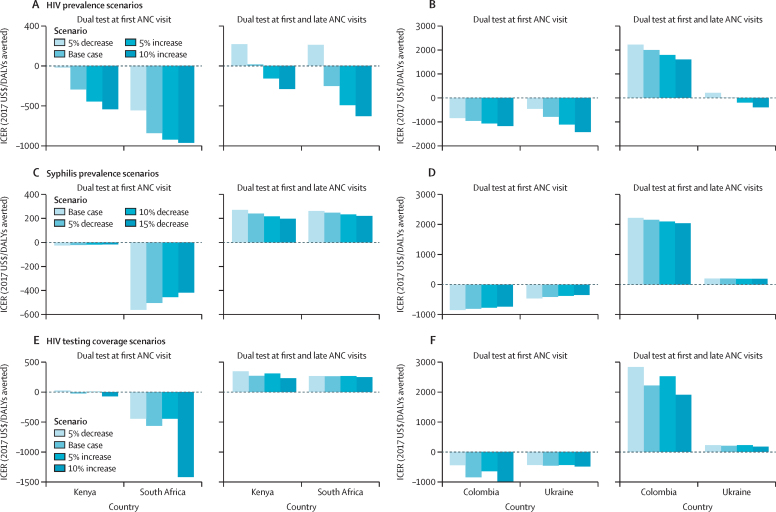
Scenario implementation analyses (A) Decreasing HIV prevalence in Kenya and South Africa. (B) Decreasing HIV prevalence in Colombia and Ukraine. (C) Decreasing syphilis prevalence in Kenya and South Africa. (D) Decreasing syphilis prevalence in Colombia and Ukraine. (E) Variation in HIV testing acceptance in Kenya and South Africa. (F) Variation in HIV testing acceptance in Colombia and Ukraine. Base case used independent tests at country-specific timepoints. Scenario one used dual HIV and syphilis testing with no retesting during late ANC. Scenario two used independent HIV and syphilis tests with retesting during late ANC. Scenario three used dual HIV and syphilis testing with retesting during late ANC. ICERs compare scenario one with the base case and scenario three with scenario one, under alternative parameter values. ANC=Antenatal care. DALY=disability-adjusted life-year. ICER=incremental cost-effectiveness ratio.

Cost-effectiveness of various strategies were also robust to decreases in maternal HIV and syphilis prevalence, and changes in HIV testing coverage across scenarios and countries (figure 2). ICERs were most sensitive to changes in HIV testing coverage and decreases in HIV prevalence in settings of intermediate (Kenya) and high (South Africa) HIV prevalence.

## Discussion

Our model-based analysis projected that incorporating a dual HIV and syphilis rapid diagnostic test at the first antenatal care visit is cost-saving in settings of high (South Africa), intermediate (Kenya), and low (Colombia and Ukraine) HIV prevalence, compared with using individual HIV and syphilis tests. Overall, our results suggest syphilis test coverage in antenatal care could increase if dual rapid diagnostic tests are used and that dual tests have the potential to overcome any limitations, such as the time to do individual HIV or syphilis tests. Conclusions were consistent across a range of settings with varied maternal syphilis prevalence (0·41–2·5%), and the results are consistent with a previous analysis from Malawi.^9^ Compared with individual tests for HIV and syphilis, dual HIV and syphilis testing at the first antenatal care visit averted congenital syphilis infections and DALYs, but it led to small increases in the absolute number of infant HIV infections in Kenya and South Africa. Increases in infant HIV infections are attributed to increased infant survival when syphilis-related stillbirth and neonatal deaths were averted, leading to a larger population of infants at risk for HIV.

In all countries, scenario three, which used a dual rapid diagnostic test at the first antenatal care visit and for retesting, was less costly and more effective than scenario two, which used individual tests at the same timepoints. Cost savings were primarily driven by lower costs per woman tested with a dual rapid diagnostic test at the first antenatal care visit compared with combined overall costs of testing with individual assays. In all countries except Kenya, a dual rapid diagnostic test kit was less expensive than the combined cost of an HIV rapid diagnostic test kit and a rapid plasma reagin test. Furthermore, testing with dual rapid diagnostic tests reduced labour costs in all countries. Although the dual rapid diagnostic test remained cost-effective with 20% increases in test kit cost, it was not always cost saving.

Because retesting did not avert additional syphilis infections in countries with intermediate and high HIV prevalence and antenatal care visits occurred after 32 weeks in these settings, retesting for HIV only (not modelled) is likely to be similarly effective and less costly than retesting with a dual rapid diagnostic test. In a previous analysis of the maternal HIV retesting model used for this analysis, retesting averted few infant HIV infections and has restricted potential for the prevention of MTCT of HIV in low HIV prevalence countries (Meisner J, unpublished data). Although retesting for maternal syphilis is not recommended by WHO, if retesting occurs early enough for maternal syphilis treatment to prevent adverse birth outcomes (≤32 weeks), it has the potential to avert stillbirths and congenital syphilis cases attributed to incident maternal syphilis infections or infections not previously detected. Our results suggest that retesting in countries with low HIV prevalence would avert few HIV infections and no congenital syphilis infections.

Operationally, retesting using dual HIV and syphilis rapid diagnostic tests might have advantages for simplifying testing algorithms that avoid laboratory-based syphilis tests and reducing prices through increasing procurement volumes in high HIV-burden settings. However, challenges exist for the allocation of restricted resources towards a costly retesting strategy with minimal benefits. Programmes will need to clarify unique needs for women previously diagnosed with HIV who only require syphilis testing, and for those diagnosed with syphilis during their current pregnancy who require only HIV testing. Although many PMTCT programmes use HIV rapid diagnostic tests, laboratory testing for syphilis is still common in many low-income and middle-income settings. The logistics of laboratory testing for syphilis—such as sample collection following antenatal care visits and delays in waiting for laboratory results (eg, confirmatory assays for reactive tests)—might result in women not getting tested, not receiving test results, lower syphilis treatment coverage, and more congenital syphilis cases.

Dual HIV and syphilis rapid diagnostic tests have many characteristics that appeal to individuals accessing services and health-care providers. They only require a single blood draw and provide immediate results, allowing health-care providers to make life-saving decisions regarding treatment more quickly.^26^ Training for health-care providers would be simplified because of the need for only one initial screening test for HIV and syphilis, rather than two. Additionally, dual rapid diagnostic tests have the advantage of streamlined procurement of test kits and reagents. However, countries will still need a supply of single HIV rapid diagnostic tests to avoid syphilis retesting when not indicated, to comply with national testing policies, to prevent missed opportunities to detect maternal HIV or syphilis infection, and to prevent MTCT in the event of dual rapid diagnostic test stock-outs. This could result in challenges determining which programmes should fund test kits, with siloed funding streams for HIV and syphilis. In addition, the sensitivity and specificity of the syphilis component of the dual assay has been reported to be variable under field conditions, and quality control measures should be followed to ensure accurate interpretation of results.^27^

Our analysis should be viewed in the context of some limitations. Syphilis testing in antenatal care might be heterogeneous between and within countries, and the assumption that countries exclusively use laboratory-based syphilis testing might be inaccurate. There could be variation in the assay type within a country based on geographical differences or programmatic partners. Our results might have restricted generalisability to settings currently using rapid diagnostic tests for syphilis, and are subject to market prices for syphilis and HIV test kits and labour costs. Our retesting scenarios include women who are tested for the first time during late antenatal care, which might overstate the benefit of the retest relative to the first test. Because of the separate construction of the HIV and syphilis models, we were unable to model syphilis and HIV co-infection, and did not model risk of fetal loss due to HIV. However, whether risks of MTCT of HIV are higher in the context of maternal co-infection is unclear.^26^, ^28^, ^29^, ^30^ Additionally, because of the small amount of data on the effectiveness of treating maternal syphilis infection after 32 weeks' gestation, we assumed that there would not be an effect on PMTCT of syphilis after this point, which might underestimate maternal and infant benefits of retesting later in pregnancy. We also restricted syphilis-related disability to the first 3 years of life, which probably underestimates the burden of congenital syphilis. Adding additional years of disability from congenital syphilis would increase the positive health effect of dual testing. Although we used the literature and in-country experts to populate model parameters, some parameters were unavailable or based on a small amount of data, particularly related to syphilis. Future studies are needed to inform assumptions about breastfeeding practices, syphilis treatment coverage, and rates of adverse maternal syphilis outcomes. Our results were qualitatively robust to all one-way sensitivity analyses, but ICER estimates might be imprecise. We had insufficient data to inform many parameter distributions and therefore did not do probabilistic sensitivity analysis to estimate credible intervals. Despite the limitations, our results are probably conservative estimates because we did not include maternal treatment benefits, such as improved quality of life, reduced risk of sexual transmission, or health system costs averted. Our estimate of time to complete a rapid test of 30 min per patient might be conservative in some settings. Our analysis has several strengths. We included countries that represented variations in HIV prevalence, syphilis prevalence, and geographical region. We compared HIV and syphilis testing strategies across countries using the same methods and model structure, and similar assumptions. Our analysis expanded on previous studies^9^ by including incident syphilis and HIV infections, in addition to prevalent infections.

Dual HIV and syphilis rapid diagnostic test as the first test in antenatal care can improve efforts to provide integrated services within the antenatal care setting, saving overall costs while improving health outcomes for women and their children. Breaking down siloes of vertical programmes can improve the quality of antenatal care and help to streamline service delivery, thereby accelerating progress on efforts to achieve dual elimination of MTCT of HIV and syphilis.

## References

[1] Korenromp EL, Rowley J, Alonso M (2019). Global burden of maternal and congenital syphilis and associated adverse birth outcomes-estimates for 2016 and progress since 2012. PLoS One.

[2] UNAIDS (2018). UNAIDS data 2018. https://www.unaids.org/sites/default/files/media_asset/unaids-data-2018_en.pdf.

[3] Alistar SS, Owens DK, Brandeau ML (2014). Effectiveness and cost effectiveness of oral pre-exposure prophylaxis in a portfolio of prevention programs for injection drug users in mixed HIV epidemics. PLoS One.

[4] WHO (2016). Global health sector strategy on sexually transmitted infections 2016–2021. http://www.who.int/reproductivehealth/publications/rtis/ghss-stis/en/.

[5] WHO (2016). Global Health Sector Strategy on HIV 2016–2021. https://apps.who.int/iris/bitstream/handle/10665/246178/WHO-HIV-2016.05-eng.pdf.

[6] WHO (2017). WHO validation for the elimination of mother-to-child transmission of HIV and/or syphilis. https://www.who.int/reproductivehealth/congenital-syphilis/WHO-validation-EMTCT/en/.

[7] UNAIDS (2020). Seizing the moment: tackling entrenched inequalities to end epidemics.

[8] Baker U, Okuga M, Waiswa P (2015). Bottlenecks in the implementation of essential screening tests in antenatal care: syphilis, HIV, and anemia testing in rural Tanzania and Uganda. Int J Gynaecol Obstet.

[9] Bristow CC, Larson E, Anderson LJ, Klausner JD (2016). Cost-effectiveness of HIV and syphilis antenatal screening: a modelling study. Sex Transm Infect.

[10] Drake AL, Thomson KA, Quinn Ca (2019). Retest and treat: a review of national HIV retesting guidelines to inform elimination of mother-to-child HIV transmission (EMTCT) efforts. J Int AIDS Soc.

[11] WHO (2019). Consolidated guidelines on HIV testing services for a changing epidemic: policy brief. https://www.who.int/publications/i/item/consolidated-guidelines-on-hiv-testing-services-for-a-changing-epidemic.

[12] Albright CM, Emerson JB, Werner EF, Hughes BL (2015). Third-trimester prenatal syphilis screening: a cost-effectiveness analysis. Obstet Gynecol.

[13] Hersh AR, Megli CJ, Caughey AB (2018). Repeat screening for syphilis in the third trimester of pregnancy: a cost-effectiveness analysis. Obstet Gynecol.

[14] Ishikawa N, Dalal S, Johnson C (2016). Should HIV testing for all pregnant women continue? Cost-effectiveness of universal antenatal testing compared to focused approaches across high to very low HIV prevalence settings. J Int AIDS Soc.

[15] WHO (2017). WHO guideline on syphilis screening and treatment for pregnant women.

[16] Department of Information Evidence and Research (2018). WHO methods and data sources for global burden of disease estimates. http://www.who.int/healthinfo/global_burden_disease/GlobalDALY_method_2000_2016.pdf.

[17] Salomon JA, Vos T, Hogan DR (2012). Common values in assessing health outcomes from disease and injury: disability weights measurement study for the Global Burden of Disease Study 2010. Lancet.

[18] Smith DH, Gravelle H (2001). The practice of discounting in economic evaluations of healthcare interventions. Int J Technol Assess Health Care.

[19] The World Bank World Development Indicators, GDP deflator. https://data.worldbank.org/indicator/NY.GDP.DEFL.ZS.

[20] Drummond MF, Sculpher MJ, Claxton K, Stoddart GL, Torrance GW (2015). Methods for the economic evaluation of health care programmes.

[21] Phillips AN, Cambiano V, Nakagawa F (2018). Cost-effectiveness of public-health policy options in the presence of pretreatment NNRTI drug resistance in sub-Saharan Africa: a modelling study. Lancet HIV.

[22] Woods B, Revill P, Sculpher M, Claxton K (2016). Country-level cost-effectiveness thresholds: initial estimates and the need for further research. Value Health.

[23] Meyer-Rath G, van Rensburg C, Larson B, Jamieson L, Rosen S (2017). Revealed willingness-to-pay versus standard cost-effectiveness thresholds: evidence from the South African HIV Investment Case. PLoS One.

[24] Topachevskyi O, Lebega O, Oleschchuk O (2018). Estimation of supply side cost effectiveness threshold in Ukraine: perspective use in health care decision-making. Value Health.

[25] Faria R, Duarte A, McKenna C, Revill P, Cary M, Byford S (2014). Guidelines for the economic evaluation of healthcare technologies in Colombia: technical support documents. https://pdfs.semanticscholar.org/f5d0/7390bf41a2cbb197e089fb56e828d9a7e589.pdf.

[26] Gliddon HD, Peeling RW, Kamb ML, Toskin I, Wi TE, Taylor MM (2017). A systematic review and meta-analysis of studies evaluating the performance and operational characteristics of dual point-of-care tests for HIV and syphilis. Sex Transm Infect.

[27] Lee M-J, Hallmark R, Frenkel L, Del Priore G (1998). Maternal syphilis and vertical perinatal transmission of human immunodeficiency virus type-1 infection. Int J Gynecol Obstet.

[28] Mwapasa V, Rogerson SJ, Kwiek JJ (2006). Maternal syphilis infection is associated with increased risk of mother-to-child transmission of HIV in Malawi. AIDS.

[29] Schulte JM, Burkham S, Hamaker D (2001). Syphilis among HIV-infected mothers and their infants in Texas from 1988 to 1994. Sex Transm Dis.

[30] Yeganeh N, Watts HD, Camarca M (2015). Syphilis in HIV-infected mothers and infants: results from the NICHD/HPTN 040 study. J Pediatric Infect Dis Soc.

