# Supplementary material for: Cost-effectiveness of dual maternal HIV and syphilis testing strategies in high and low HIV prevalence countries: a modelling study
Source: Lancet Glob Health. 2020 Nov 20;9(1):e61–71. doi: 10.1016/S2214-109X(20)30395-8 (PMC7783487; doi:10.1016/S2214-109X(20)30395-8)
Supplement: Supplementary appendix [file mmc1.pdf]

THE LANCET
Global Health

Supplementary appendix

This appendix formed part of the original submission and has been peer reviewed. We post it as supplied by the authors.

Supplement to: Rodriguez PJ, Allen RobertsD, Meisner J, et al. Cost-effectiveness of dual maternal HIV and syphilis testing strategies in high and low HIV prevalence countries: a modelling study. *Lancet Glob Health* 2020; published online Nov 20. http://dx.doi.org/10.1016/S2214-109X(20)30395-8.

Appendix 1

Figure A1a: **Schematic of the Markov cohort model.** Maternal HIV disease states (A-F) were constructed based on the following: HIV status (one negative, five positive), ability for assay to detect p24 antigen or HIV antibodies (Fiebig Stages I/II vs III), known vs. unknown HIV positive status, and use of ART. Pregnancy/postpartum stages were based on maternal and child health (MCH) visit schedules for antenatal care (ANC), facility delivery, and postnatal care/infant immunizations. Pregnancy/postpartum stages were modeled to occur between the following time periods: 0=onset of pregnancy to first ANC, 1=first to second ANC, 2=second ANC to delivery, 3=delivery (one week period), 4=one to six weeks postpartum, 5=six to 14 weeks postpartum, 6=14 weeks to six months postpartum, 7=six to nine months postpartum, and 8=nine to 12 months postpartum. Transitions across columns correspond to within stage (between visit) transitions, while transitions across rows correspond to across stage (at visit) transitions. Women can transition from any state to death (not shown in the model). Ag+ = p24 antigen positive (Fiebig stages I/II); Ab+ = antibody positive (Fiebig stage III); nART = not on ART; uk_nART = unknown HIV status, not on ART; k_nART = known HIV status, not on ART.

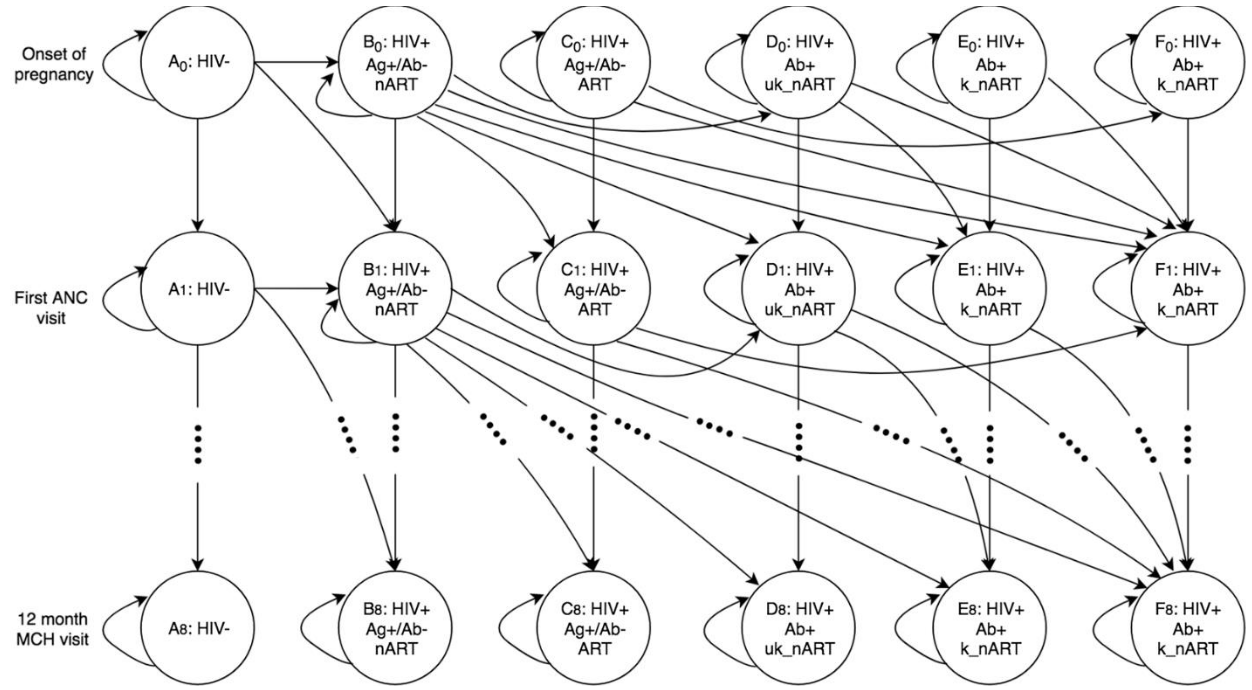

Figure A1b. **Schematic of the Markov cohort model for syphilis retesting**. Transitions are time varying. Not shown: women can transition from any state to death, an absorbing state.

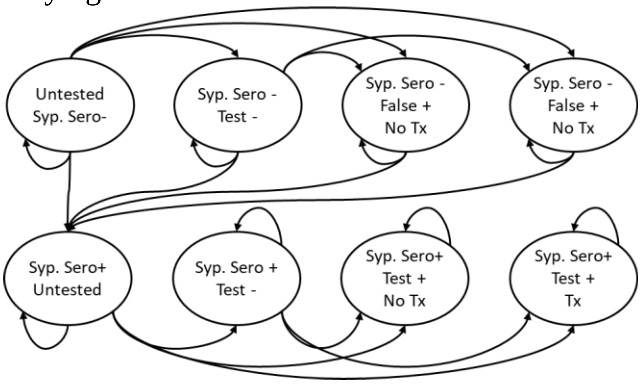

Appendix 2: Model Equations

A HIV Model Equations

1 HIV Maternal state transitions

1.1 Transition probability matrices (states)

		To					
		A	B	C	D	E	F
From	A: HIV−	$(1 - I_x)$	I_x	0	0	0	0
	B: aHIV+, $_n$ART	0	$\left(1 - \frac{1}{d_a}\right)$	0	$\left(\frac{1}{d_a}\right)$	0	0
	C: aHIV+, ART	0	$1 - ret$	$\left(1 - \frac{1}{d_a}\right) * ret$	0	0	$\left(\frac{1}{d_a}\right) * ret$
	D: cHIV+, uk$_n$ART	0	0	0	1	0	0
	E: cHIV+, k$_n$ART	0	0	0	0	1	0
	F: cHIV+, ART	0	0	0	0	$1 - ret$	ret

Table A2a: Within stage (between visit) transition probability matrix. All transition probabilities are also multiplied by $(1 - \mu_w)$ in stages 0-2 and 5-7, or by $(1 - \mu_m)$ in stages 3 and 4, whereμ_w is the weekly mortality rate of women of reproductive age, and μ_m is the weekly maternal mortality rate at parturition and early post-partum. Stage 0: onset of pregnancy to first ANC; stage 1: first ANC to second ANC; stage 2: second ANC to delivery; stage 3: delivery; stage 4: first 6 weeks postpartum; stage 5: six to 14 weeks postpartum; stage 6: 14 weeks to six months postpartum; stage 7: six to nine months postpartum; stage 8: nine to 12 months postpartum. Abbreviations: $aHIV$ = recent HIV infection, $cHIV$ = established HIV infection, uk = unknown HIV status, k = known HIV status, $_nART$ = not receiving antiretroviral treatment (ART),ART = receiving ART,I_x= weekly maternal HIV incidence in model period x, d_a= duration of recent infection, ret = proportion of women retained on ART each week.

To							
		A: HIV-	B: aHIV+, $_nART$	C: aHIV+, ART	D: cHIV+, uk$_n$ART	E: cHIV+, k$_n$ART	F: cHIV+, ART
From	A: HIV−	$(1-I_x)$	I_x	0	0	0	0
	B: aHIV+, $_nART$	0	$\left(1-\frac{1}{d_a}\right)*(1-a_n ts_n s_a rA)$	$\left(1-\frac{1}{d_a}\right)*(a_n ts_n s_a rA)$	$\left(\frac{1}{d_a}\right)*(1-a_n ts_n s_c r)$	$\left(\frac{1}{d_a}\right)*(a_n ts_n s_c r)*(1-A)$	$\left(\frac{1}{d_a}\right)*(a_n ts_n s_c r)*A$
	C: aHIV+, ART	0	0	$\left(1-\frac{1}{d_a}\right)$	0	0	$\left(\frac{1}{d_a}\right)$
	D: cHIV+, uk$_n$ART	0	0	0	$(1-a_n ts_n s_c r)$	$(a_n ts_n s_c r)*(1-A)$	$(a_n ts_n s_c r)*A$
	E: cHIV+, k$_n$ART	0	0	0	0	1	0
	F: cHIV+, ART	0	0	0	0	0	1

Table A2b: Between stage (at visits) transition probability matrix. All transition probabilities are also multiplied by $(1-\mu_w)$ in stages 0-2 and 5-8, or by $(1-\mu_m)$ in stages 3 and 4. Stage 0: onset of pregnancy to first ANC; stage 1: first ANC to second ANC; stage 2: second ANC to delivery; stage 3: delivery; stage 4: first 6 weeks postpartum; stage 5: six to 14 weeks postpartum; stage 6: 14 weeks to six months postpartum; stage 7: six to nine months postpartum; stage 8: nine to 12 months postpartum. Abbreviations: $aHIV$ = recent HIV infection, $cHIV$ = established HIV infection, uk = unknown HIV status, k = known HIV status, $_nART$ = not receiving antiretroviral treatment (ART), ART = receiving ART, I_x= weekly maternal HIV incidence in model period x, and d_a= duration of recent infection, a_n= visit attendance at stage n (ANC visit, MCH visit, or facility delivery), ts_n = probability of being tested at visit corresponding to stage n, s_a= sensitivity of rapid screening test in recent infection, s_c= sensitivity of rapid screening test in established infection, A = probability of accepting ART if confirmed HIV positive, r = probability of receiving results if testing positive.

1.2 Derived parameters

Maternal HIV incidence in model period x is derived by:

(1.1) $I_x = incidence_x * [1 - (red_{prep} * p_{prep})]$

Where $incidence_x$is weekly maternal HIV incidence in model period x, red_{prep} is the expected reduction in incidence due to pre-exposure prophylaxis (PrEP) use, andp_{prep} is the probability of fully-adherent PrEP use.

The probability of being tested at the visit corresponding to stage n is derived by:

(1.2) $ts_n = (test_{prob} * (1 - stockout) * test_{accept})$

Where $test_{prob}$is the probability of the test being offered at the visit corresponding to stage n (set by the model user), $stockout$is the probability of test kit stockout, and $test_{accept}$is the probability the patient consents to testing. At delivery (n=3), ts_3 is multiplied by $1 - ts_2$ to reflect that testing occurs at late gestation (second ANC, n=2), not both. Similarly, at six weeks postpartum (n=4), ts_4 is multiplied by $1 - ((1 - ts_2) * ts_3)$ to reflect that testing only occurs at six weeks postpartum only if it did not occur at the previous two time points.

The duration of recent infection is derived by:

(1.3) $d_a = d_{Ag-} + d_{Ag+/Ab-} + d_{Ab+/hVL}$

Where $d_{Ag-}$is the duration of the antigen-negative period, $d_{Ag+/Ab-}$is the duration of the antigen-positive, antibody-negative period, and $d_{Ab+/hVL}$ is the duration of the antibody-positive (post-seroconversion) period before set-point viral load is reached.

All other parameters are defined as detailed in Appendix B: Model Parameters.

2 HIV Starting states (stage 0)

No women are assumed to be in state C (HIV positive, recent infection, on ARTs) at the start of the model period.

2.1 State A (HIV negative)

The number of women who start their pregnancy (and thus the model period) in State A is given as

(2.1) $A_0 = N[(1 - prev_{preg}) - \left(I_{early_{preg}} * d_a\right)]$

Where $prev\_preg$ is the prevalence of HIV among women at the onset of pregnancy, and N is the total population size.

2.2 State B (HIV positive, recent infection, not on ARTs)

The number of women who their pregnancy in State B is given as

(2.2) $B_0 = I_{early\_preg} * d_a * N$

Where I_{early_preg} is the weekly incidence of HIV among women in early pregnancy, and d_a is the duration of recent infection.

2.3 State D (HIV positive, established infection, not on ARTs, unknown status)

The number of women who start their pregnancy in State D is given as

(2.3) $D_0 = prev\_preg * (1 - status\_known) * N$

Where $status\_known$ is the probability an HIV positive woman at the start of pregnancy knows her status.

2.4 State E (HIV positive, established infection, not on ARTs, known status)

The number of women who start their pregnancy in State E is given as

(2.4) $E_0 = prev\_preg * status\_known * (1 - A) * N$

Where A is the probability of accepting ART if confirmed HIV positive.

2.5 State F (HIV positive, established infection, on ARTs)

The number of women who start the model period in State F is given as

(2.5) $F_0 = prev\_preg * status\_known * A * N$

Where A is the probability of accepting ART if confirmed HIV positive.

3 MTCT of HIV

Potential mother-to-child transmission of HIV is assumed to occur from all women in an HIV positive state (states B-F). For all equations that follow, S indexes state (A-F), and n indexes stage (0-8). For instance X_{F2} refers to variable X pertaining to state F (established infection, on ARTs), stage 2 (after second ANC, prior to delivery). Maternal deaths at each time step enter an absorbing state, to which none of the following equations are applied. This model assumes no miscarriages occur and that all pregnancies are singleton. To reflect time from testing to ART initiation as well as time from ART initiation to viral suppression, the model assumes no viral suppression among mothers with recent HIV infection on ART.

3.1 *In utero* (stages 0-2)

The number of infant infections generated by women in states B or C($S \in \{B, C\}$)at stage n of pregnancy($n \in \{0,1,2\}$)is given as

(3.1.1) $Inf_{utero\_Sn} = \left(1 - e^{-\lambda_{utero\_a} * t_n}\right) * (N_{Sn} - Inf_{Sn-1}); S \in \{B, C\}, n \in \{0,1,2\}$

Where $\lambda_{utero_a}$is the weekly incidence of *in utero* infant transmission from a recently infected mother, $t_n$is duration of stage n (in person-weeks),N_{Sn} is the number of women in stage n and state S, and Inf_{Sn-1} is the number of infants infected in the same state S but the previous stage (n-1).

The number of infant infections generated by women in states D or E at stage n of pregnancy($n \in \{0,1,2\}$)is given as

(3.1.2) $Inf_{utero\_Sn} = \left(1 - e^{-\lambda_{utero\_c} * t_n}\right) * (N_{Sn} - Inf_{Sn-1}); S \in \{D, E\}, n \in \{0,1,2\}$

Where $\lambda_{utero_c}$is the weekly incidence of *in utero* infant transmission from a mother with an established infection.

The number of infant infections generated by women in state F at stage n of pregnancy($n \in \{0,1,2\}$) is given as

(3.1.3) $Inf_{utero\_Sn} = \left(1 - e^{-(\lambda_{utero\_c}[1-(V_{red} * p_{VL})]) * t_n}\right) * (N_{Sn} - Inf_{Sn-1}); S \in \{F\}, n \in \{0,1,2\}$

Where V_{red} is the proportional reduction in infant transmission incidence attributable to maternal viral load suppression (undetectable viral load), and$p_{VL}$is the probability that a woman receiving ARTs has achieved viral load suppression.

3.2 Delivery and early post-partum (stages 3 and 4)

The number of infant infections generated by women in states B or D at delivery (parturition; n=3) or the first six weeks postpartum (n=4) is given as

(3.2.1) $Inf_{part\_Sn} = \left(1 - e^{-(\lambda_{part\_y} * t_n)}\right) * (N_{Sn} - Inf_{Sn-1}) * (1 - \mu_{ne}); S \in \{B, D\}, n \in \{3,4\}$

Where λ_{part_y} is the weekly incidence of infant transmission at parturition and early post-partum from a mother with recent infection if y=a, and established infection if y=c; $t_n$is the duration of model stage n in person-weeks; and$\mu_{ne}$is the probability of neonatal death.

The number of infant infections generated by women in states C or E at delivery (parturition; n=3) is given as

(3.2.2) $Inf_{part\_Sn} = \left(1 - e^{-(\lambda_{part\_y} * [1-(ARV_{red} * p\_ARV)] * t_n)}\right) * (N_{Sn} - Inf_{Sn-1}) * (1 - \mu_{ne}); S \in \{C, E\}, n \in \{3\}$

Where $ARV_{red}$is the proportional reduction in infant transmission incidence attributable to infant antiretroviral (ARV) prophylaxis, and$p_ARV$is the probability the infant recieves ARV prophylaxis.

The number of infant infections generated by women in states C or E at during the first six weeks postpartum (n=4) is given as

(3.2.3) $$Inf_{part\_Sn} = \left(1 - e^{-(\lambda_{part\_y} * [1-(ARV_{red} * p\_ARV)] * [1-(nbf_{red} * p\_nbf_e)] * t_n)}\right) * (N_{S4} - Inf_{S4-1}) * (1 - \mu_{ne}); S \in \{C, E\}, n \in \{4\}$$

Where$nbf_{red}$is the proportional reduction in infant transmission incidence attributable to not breastfeeding andp_nbf_e is the probability of complete breastfeeding avoidance in the early postpartum period.

The number of infant infections generated by women in state F at delivery (parturition; n=3) is given as

(3.2.4) $$I_{part\_Sn} = \left(1 - e^{-(\lambda_{part\_c}*[1-(ARV_{red}*p\_ARV)]*[1-(V_{red}*p_{VL})]*t_n)}\right) * (N_{Sn} - I_{Sn-1}) * (1 - \mu_{ne});$$
$$S \in \{F\}, n \in \{3\}$$

And the number of infant infections generated by women in state F during the first six weeks postpartum (n=4) is given as

(3.2.5) $$I_{part\_Sn} = \left(1 - e^{-(\lambda_{part\_c}*[1-(ARV_{red}*p\_ARV)]*[1-(V_{red}*p_{VL})]*[1-(nbf_{red}*p\_nbf_e)]*t_n)}\right)$$
$$* (N_{Sn} - I_{Sn-1}) * (1 - \mu_{ne}); S \in \{F\}, n \in \{4\}$$

3.3 Mid and late post-partum (stages 5-8)

The number of infant infections generated by women in states B or D and stage n (n>4) post-partum is given as

(3.3.1) $$Inf_{pp\_Sn} = \left(1 - e^{-(\lambda_{pp\_x\_y}*[1-(nbf_{red}*p\_nbf_{xk})]*t_n)}\right) * (N_{Sn} - Inf_{Sn-1}) * (1 - \mu_z);$$
$$S \in \{B, D\}, n \in \{5,6,7,8\}$$

Where $\lambda_{pp\_x\_y}$ is the weekly incidence of infant transmission in model period x (x=m, mid postpartum; x=l, late postpartum) from a mother who is exclusively breastfeeding and has recent infection if y=a, or established infection if y=c;p_nbf_x is the probability of complete breastfeeding avoidance in model period x, HIV status k (no known infection= states A, B, and D; known infection= states C, E, and F);$\mu_z$is the probability of neonatal (z=ne, up to 6 weeks post-partum) or infant (z=i, 6 weeks to 12 months post-partum) death.

The number of infant infections generated by women in states C or E and stage n (n>4) post-partum is given as

(3.3.2) $$Inf_{pp\_Sn} = \left(1 - e^{-(\lambda_{pp\_x\_y}*[1-(ARV_{red}*p\_ARV)]*[1-(nbf_{red}*p\_nbf_{xk})]*t_n)}\right) *$$
$$(N_{Sn} - Inf_{Sn-1}) * (1 - \mu_z); S \in \{C, E\}, n \in \{5,6,7,8\}$$

The number of infant infections generated by women in state F and stage n (n>4) post-partum is given as

(3.3.3) $$Inf_{pp\_Sn} = \left(1 - e^{-(\lambda_{pp\_x\_c}*[1-(ARV_{red}*p\_ARV)]*[1-(nbf_{red}*p\_nbf_{xk})]*[1-(V_{red}*p_{VL})]*t_n)}\right)$$
$$* (N_{Sn} - Inf_{Sn-1}) * (1 - \mu_z); S \in \{F\}, n \in \{5,6,7,8\}$$

Note that the user does not have access to parameters that allow disaggregation of breastfeeding practice by HIV status or doesn't feel this is appropriate, they may enter the same probability of breastfeeding practice across HIV positive and HIV negative women, effectively collapsing over the k subscript for p_nbf_{xk}.

4 HIV Testing and Treatment Costs

4.1 Maternal testing costs

Testing is assumed to be linked to ANC and MCH visits that would occur in the absence of testing, thus only incremental costs associated with testing at a given visit are modeled. Testing occurs only from negative/unknown states (A, B, and D).

4.1a All negative/unknown status states

Total costs for screening women at stage n independent of HIV status is given as

(4.1.1) $C_{screen\_Sn} = a_n * ts_n * screen * N_{test\_\tau_{1n}}; S \in \{A, B, D\}, n \in \{1,2, \ldots ,8\}$

Where $screen$ is the total cost of screening using test per woman tested, and $N_{test\_\tau_{1n}}$ is the number of women in the test set at time step τ_{1n}.

Inputs for the total cost of a screening test may include:

- Test kit costs
- Other supplies including gloves, capillary tubes, alcohol swabs, etc.
- Staff and administrative costs above that required for the existing visit (ANC, delivery, or PNC)

4.1b HIV negative women (State A)

Additional testing costs for HIV negative women at stage n are given as

(4.1.2) $C_{false\_pos\_Sn} = a_n * ts_n * (1 - spec) * false\_pos * N_{A\_\tau_{1n}}; S \in \{A\}, n \in \{1,2, \ldots ,8\}$

Where $spec$ is the specificity of the screening test, $N_{A\_\tau_{1n}}$ is the number of women in state A at time step τ_{1n}, and $false\_pos$ is the cost of a false positive test above that incurred for screening.

Inputs for cost of a false positive test may include:

- Confirmatory and tie-breaker testing
- Additional staff and facility costs

Thus, total testing costs for women in state A at stage n are given as:

(4.1.3) $C_{Sn} = a_n * ts_n[screen + ((1 - spec) * false\_pos)] * N_{A\_\tau_{1n}}; S \in \{A\}, n \in \{1,2,..,8\}$

4.1c HIV positive women (States B and D)

Additional costs for HIV positive women are given as:

(4.1.4) $C_{true\_pos\_Sn} = a_n * ts_n * sens * r * true\_pos * N_{S\_\tau_{1n}}; S \in \{B, D\}, n \in \{1,2,..,8\}$

Where $N_{S\_\tau_{1n}}$ is the number of women in state S at time step τ_{1n}, and $true\_pos$ is the cost of a true positive test above that incurred for screening.

Inputs for the cost of a true positive test may include:

- Confirmatory and tie-breaker testing
- Staff and facility costs associated with additional counseling and linkage to care

Additionally, women newly diagnosed at stages 3 (delivery) or later are offered infant prophylaxis at the time of their diagnosis.

Thus, the total costs for women in states B or D at stage n where n <3 are given as:

(4.1.5) $\mathrm{C}_{Sn} = a_n * ts_n[screen\mathrm{n} + (sens * r * true\_pos)] * N_{S\_\tau_{1n<3}}; S \in \{B, D\}, n \in \{0,1,2\}$

And, the total costs for women in states B or D at stage n where n ≥ 3 are given as:

(4.1.6) $$\mathrm{C}_{Sn} = a_n * ts_n[screen\mathrm{n} + (sens * r * (true\_pos + (\mathrm{cost}_{ARV} * p\_ARV))] * N_{S\_\tau_{1n\geq 3}}; \quad S \in \{B, D\}, n \in \{3,4,\ldots,8\}$$

Where $cos\,t_{arv}$is the total cost of a full course of infant prophylaxis (the cost of an entire is course applied at one time, not per week).

4.1d Women in a known status state (C, E, and F)

At delivery (stage n=3) women with known HIV infection (states C, E, and F) are offered ARVs for infant prophylaxis. Women in states C and F also incur costs associated with maternal ART.

Thus, total costs for women in state C or F at stage n≠ 3 are given as:

(4.1.7) $C_{Sn} = cos\,t_{ART} * N_{Sn_{\neq 3}} * t_{n\neq 3}; S \in \{C, F\}, n \in \{1,2,4,\ldots,8\}$

Where $cos\,t_{art}$ is the total cost of maternal ART per week,$N_{Sn_{\neq 3}}$is the number of women state S and stage n≠ 3, and $t_{n\neq 3}$ is the duration of stage n (where n≠3) in weeks.

Total costs for women in state C or F at stage n=3 are given as:

(4.1.8) $\mathrm{C}_{Sn} = [(\mathrm{cost}_{ARV} * p\_ARV) + (\mathrm{cost}_{ART} * p\_ART)] * N_{Sn} * t_n; S \in \{C, F\}, n \in \{3\}$

And total costs for women in state E at stage n=3 are given as:

(4.1.9) $\mathrm{C}_{Sn} = (\mathrm{cost}_{ART} * p\_ARV) * N_{Sn} * 1; S \in \{E\}, n \in \{3\}$

Note, women in state E do not incur costs at any other time stages.

4.2 Maternal pre-exposure prophylaxis (PrEP) costs

Women in states A and B are also eligible to receive PrEP. PrEP costs for women in states A and B at stage n are given as:

(4.2.1) $\mathrm{C}_{PrEP\_Sn} = 0 \leq n \leq 8: (\mathrm{cost}_{\mathrm{Pr}EP} * p\_prep) * N_{Sn} * t_n; S \in \{A, B\}, n \in \{1,2,\ldots,8\}$

Where $cos\,t_{Pr\,EP}$is the per-week cost of pre-exposure prophylaxis;$p_prep$is the probability of PrEP reciept and adherence;$N_{Sn}$is the number of women in state S at stage n; and $t_n$is the duration of stage n in weeks.

4.3 Pediatric tretment costs

Pediatric treatment costs were calculated over a 20-year time horizon (from birth through age 19) assuming an annual discount rate of 3%.

For an HIV-infected child of age a, $a \in \{1, \ldots, 19\}$ the annual cost of ART was calculated as follows:

(4.3.1) $C_{i\_a} = \frac{cost_{ART} * p_{ART\_a} * 52}{(1+r)^{a+1}}$

Where $cost_{ART}$ is the weekly cost of ART, p_{ART_a} is the probability that an infected child of age a receives ART, and r is the discount rate.

For an HIV-infected infant under one year of age infected in stage k, $k \in \{1,2, \ldots 8\}$ the annual cost of ART was calculated as follows:

(4.3.2) $C_{i\_0\_k} = \frac{p_k * cost_{ART} * p_{ART\_a} * 52}{(1+r)^{a+1}}$

Where p_k is the proportion of the infant's first year of life remaining after infection in stage k (e.g., 1 for infants infected during pregnancy, 0.5 for infants infected at 6 months).

The total HIV treatment costs over a 20-year time horizon for infants infected through mother-to-child transmission were calculated as:

(4.3.3) $C_{i\_tot} = \sum_{k=1}^{8} C_{i\_0\_k} N_{i\_0\_k} + \sum_{a=1}^{19} C_a N_{i\_a}$

Where $N_{i\_0\_k}$ indicates the number of HIV-infected infants infected in stage k and N_{i_a} indicates the number of HIV-infected children alive at age a.

B Syphillis Model Equations

5 Syphillis Maternal state transitions

5.1 Transition probability matrices

		To							
		A: Sero-, Untested	B: Sero-, Test-	C: Sero-, Test+, No tx	D: Sero-, Test+, tx	E: Sero+, Untested	F: Sero+, Test -	G: Sero+, Test +, No tx	H: Sero+, Test +, Tx
From	A	$(1-I_x)$	0	0	0	I_x	0	0	0
	B	0	$(1-I_x)$	0	0	I_x	0	0	0
	C	0	0	$(1-I_x)$	0	I_x	0	0	0
	D	0	0	0	$(1-I_x)$	I_x	0	0	0
	E	0	0	0	0	1	0	0	0
	F	0	0	0	0	0	1		
	G	0	0	0	0	0	0	1	0

	H	0	0	0	0	0	0	0	1

Table A2c: Between visit transition probability matrix. All transition probabilities are also multiplied by $(1-\mu_w)$ up to delivery and $(1-\mu_m)$ at, whereμ_w is the weekly mortality rate of women of reproductive age, and μ_m is the weekly maternal mortality rate at parturition and early post-partum.

		To							
		A: Sero-, Untested	B: Sero-, Test-	C: Sero-, Test+, No tx	D: Sero-, Test+, tx	E: Sero+, Untested	F: Sero+, Test -	G: Sero+, Test +, No tx	H: Sero+, Test +, Tx
From	A	$(1-I_x)*(1-a_n c_s)$	$(1-I_x)*a_n c_s sp_s$	$(1-I_x)**a_n c_s*(1-sp_s)*(1-tx_s)$	$(1-I_x)*a_n c*(1-sp_s)*tx_s$	$I_x(1-a_n c)$	0	0	0
	B	0	$(1-I_x)*(1-a_n c_s sp_s)$	$(1-I_x)**a_n c_s*(1-sp_s)*(1-tx_s)$	$(1-I_x)*a_n c*(1-sp_s)*tx_s$	I_x	0	0	0
	C	0	0	$(1-I_x)*(1-a_n c_s)$	0	I_x	0	0	0
	D	0	0	0	$(1-I_x)*(1-a_n c_s)$	I_x	0	0	0
	E	0	0	0	0	$(1-a_n c_s)$	$a_n c_s*(1-se_s)$	$a_n c_s se_s*(1-tx_s)$	$a_n c_s se_s tx_s$
	F	0	0	0	0	0	$(1-a_n c_s)*(1-se_s)$	$a_n c_s se_s*(1-tx_s)$	$a_n c_s se_s tx_s$
	G	0	0	0	0	0	0	1	0
	H	0	0	0	0	0	0	0	1

Table A2d: At visit transition probability matrix. All transition probabilities are also multiplied by $(1-\mu_w)$ up to delivery and $(1-\mu_m)$ at delivery, whereμ_w is the weekly mortality rate of women of reproductive age, and μ_m is the weekly maternal mortality rate at parturition and early post-partum. Not shown, a one-week delay in test results occurs under lab-based testing, modeled as a tunnel state between untested and tested states. Among syphilis negative women, incident infection can occur while awaiting results. Women who previously received a false positive diagnosis (states B & C) can have incident infection, shown in B-E and C-E transitions above, but are ineligible for repeat testing given previous positive diagnosis. These women move to a separate sub-state of E, E.1 (not shown for simplicity), to distinguish them from women eligible for retesting. Abbreviations: I_x= weekly maternal syphilis incidence in model step x, a_n= visit attendance at visit n (first ANC or late ANC) c_s = test coverage for scenario s (individual or dual testing at first ANC or first and late ANC) se_s = sensitivity for test in scenario s (individual tests or dual RDT), sp_s = specificity for test in scenario s, tx_s= probability of receiving treatment for scenario s.

6 Syphillis starting states

All women begin in state A (syphillis -, untested) or E (syphillis positive, untested).

The number of women who their pregnancy in State A is given as:

(6.1) $A_0 = N(1-prev_{preg})$

The number of women who their pregnancy in State E is given as:

(6.2) $E_0 = N(prev_{preg})$

Where N is the number of annual pregnancies and $(prev_{preg})$ is the prevalence of syphilis in pregnancy.

7 MTCT of Syphilis

7.1 Active versus Past Maternal Syphilis

Adverse infant outcomes are modeled at birth. We assume syphilis-related adverse outcomes occur only in women who had an active syphilis infection. We therefore exclude women who tested positive due to past infection. The number of women with active syphilis is modeled as:

(7.1.1) $N_{act} = N_{Sg} * f; S \in \{E, F, G\}, g \in \{39\}$

Where N_{Sg} refers to the number of women in syphilis infection states, S ∈ (E, F,G), at time of delivery, g = 39), and f refers to a test correction factor to account for the fact that not all reported syphilis cases from a given country correspond to active syphilis infections. Given heterogeneity in tests used for reporting, we used the midpoint between the correction factors for reported cases based on treponemal tests and non-treponemal tests, respectively: $0.529 = (0.522 + 0.536)/2$. (Ham, 2015).

7.2 Adverse Birth Outcomes

The number of adverse birth outcomes were calculated as:

(7.2.1) $O_q = N_{act,ntx} * p_{q,ntx} + N_{act,tx} * p_{q,tx}$

Where O_q is the number of infants with outcome $q \in \{clinical\ congenital\ syphillis, stillbirth, neonatal\ death, low\ birth\ weight, asymptomatic\ syphillis\}$, $N_{act,ntx}$ and $N_{act,tx}$ are the number of women with active syphilis infection who are untreated and treated, respectively, and $p_{q,ntx}$ and $p_{q,tx}$ are probabilities of each outcome q amoung infants of untreated and treated mothers, respectively. Women treated after 32 weeks of gestation were assumed to have the same outcome rates as untreated women ($p_{q,ntx}$).

8 Syphillis Testing and Treatment Costs

8.1 Maternal testing costs

Testing is assumed to be linked to ANC and MCH visits that would occur in the absence of testing, thus only incremental costs associated with testing at a given visit are modeled. Testing occurs only from the untested states (A, E) and previously test negative states (B,F). Additional costing detail is provided in Appendix 3.

8.1a Intital testing costs

Initial testing costs apply to all states where syphilis status is unknown or previously negative (A,B,E,F). Initial testing costs are calculated as:

(8.1.1) $C_{screen} = a_n * ts_n * N_{Sg} * screen; S \in \{A, B, E, F\}$

Where , a_n= visit attendance at visit n (first ANC, late ANC), ts_n = probability of being tested for syphilis at visit n, $screen$ is the total cost of screening using test per woman tested using either RPR or dual RDT, and N_{Sg} is the number of women in syphilis infection states, $S \in (A, B, E, F)$, at the time step corresponding to visit n.

8.1b Confirmatory testing costs

In individual testing scenarios, a positive RPR test is confirmed by a TPHA test. The TPHA confirmatory testing costs are calculated for false positives and true positives.

For false positives:

(8.1.2) $C_{screen} = a_n * ts_n * N_{Sg} * (1 - spec) * TPHA; S \in \{A, B\}$

For true positives:

(8.1.3) $C_{screen} = a_n * ts_n * N_{Sg} * sens * TPHA; S \in \{E, F\}$

Where $spec$ is the specificity of RPR tests, $sens$ is the sensitivity of RPR tests, and $TPHA$ is the additional cost of a TPHA test, which includes test kit and labor.

8.2 Maternal treatment costs

Women testing positive for syphilis are treated with penicillin. The costs of treatment are estimated as:

(8.2.1) $C_{tx} = N_+ * p_{tx} * pen$

Where C_{tx} is the total treatment cost, N_+ is the number of women testing positive (including true and false positives), p_{tx} is the probability of treatment given a test positive result, and pen is per treatment penicillin cost.

8.3 Infant treatment costs

We assumed infants born with congenital syphilis to mothers with a positive test (regardless of treatment) would be treated in an inpatient setting for 10 days with 10M units of intravenous benzyl penicillin. Infant treatment costs are calculated as:

(8.3.1) $C_{Itx} = O_{cs} * (10 * IPbed + IVpen); S \in \{G, H\}$

Where O_{cs} is the number of infants with clinical congenital syphilis, $IPbed$ is the cost per inpatient bed day, $IVpen$ is the cost of 10M units of benzyl penicillin treatment.

C Total Infant Outcomes

9 Unique Infant Infections

HIV and syphilis were modeled separately, each with a full population. To account for overlap between the two models, we calculated unique infections as follows.

9.1 Infants with HIV infection only

(9.1.1) $N_{hiv} = (O_{hiv}) - (1 - p_{syp} * p_{hiv})$

Where O_{hiv} is the number of total infant HIV infections, p_{syp} is the proportion of infants with congenital syphilis and p_{hiv} is the proportion of infants with HIV. $(p_{syp} * p_{hiv})$ represents infants with coinfection; this relation assumes that likelihood of congenital syphilis and HIV among infants are independent.

9.2 Infants with congenital syphillis only

(9.2.1) $N_{syp} = (O_{cs} + O_{as}) - (1 - p_{syp} * p_{hiv})$

Where O_{cs} and O_{as} are the number of infants with clinical congenital syphilis and asymptomatic syphilis, respectively.

9.3 Infants with syphilis and HIV coinfection

(9.3.1) $N_{coinf} = (O_{cs} + O_{as} + O_{hiv}) * p_{syp} * p_{hiv}$

Where N_{coinf} is the number of coinfected infants.

9.4 Infants with low birth weight

(9.4.1) $N_{lbw} = O_{lbw}$

Where N_{lbw} = the number of infants with low birth weight and O_{lbw} is the number of total infant LBW attributable to syphilis.

10 Infant Deaths

10.1 Mortality probabilities

The probability of HIV-specific death from age a to a+n conditional on surviving to age a was calculated as follows:

(10.1.1) ${}_nq_{a\_hiv\_s} = (1 - p_{ART} * p_{adh}) * {}_nq_{a\_hiv\_ntx}$

Where ${}_nq_{a\_hiv\_ntx}$ is the probability of HIV-specific death without ART from age a to a+n conditional on surviving to age a, and p_{adh} is the proportion of HIV-infected children on treatment who are adherent to ART. ${}_nq_{a\_hiv\_ntx}$ was derived from cumulative mortality estimates of 32.5% by age 1 and 52.5% by age 2 among HIV-infected infants not receiving ART(1). We apply HIV-specific mortality to infants infected with HIV only and to infants with co-infection. We assumed no additional excess mortality related to syphillis infection (beyond stillbirth and neonatal death, described below). We assumed no additional HIV-specific mortality after age 2.

The total probability of death from age a to a+n among children infected with HIV was calculated as:

(10.1.2) ${}_nq_{a\_hiv} = 1 - (1 - {}_nq_{a\_hiv\_s}) * (1 - {}_nq_{a_{nhiv}})$

Where ${}_nq_{a_{nhiv}}$ is the probability of non-HIV death from age a to a+n conditional on surviving to age a and was derived from country-specific WHO life tables (2). Note that ${}_nq_{a_{nhiv}} = 0$ for HIV-uninfected children.

Finally, the annual mortality probabilities for HIV-infected and HIV-uninfected children was calculated as follows:

(10.1.3) ${}_1q_{a\_hiv} = 1 - \exp\{\frac{\ln(1 - {}_nq_{a\_hiv})}{n}\}$

(10.1.4) ${}_{1}q_{a\_nhiv} = 1 - \exp\{\frac{\ln(1 - {}_{n}q_{a\_nhiv})}{n}\}$

Where n indicates the length of the age intervals defined in the WHO life tables (e.g., ${}_{4}q_{1}$ corresponds to an interval length of n=4).

10.2 Infant Deaths

Survival over a 20-year time horizon is modeled conditional on HIV and syphilis status at birth. Syphilis infection is assumed to confer risk of infant stillbirth and neonatal death within 28 days of delivery. Afterwards, the model assumes no additional mortality risk from congenital syphilis infection.

(10.2.1) $D_{0_{syp}} = O_{sb_{syp}} + O_{nd_{syp}}$

Where $D_{0_{syp}}$ refers to excess syphilis-related infant stillbirth and neonatal death occurring within 28 days of delivery. $O_{sb_{syp}}$ and $O_{nd_{syp}}$ refer to the number of excess stillbirth and neonatal death, respectively.

At a specific age $a \in \{0, \dots 19\}$, the number of children alive is given as follows:

(10.2.2) $N_{hiv_a} = N_{hiv_{a-1}} - N_{hiv_{a-1}} * {}_{1}q_{a-1\_hiv}$
(10.2.3) $N_{coinf_a} = N_{coinf_{a-1}} - N_{coinf_{a-1}} * {}_{1}q_{a-1\_hiv}$
(10.2.4) $N_{syp_a} = N_{syp_{a-1}} - N_{syp_{a-1}} * {}_{1}q_{a-1\_nhiv}$
(10.2.5) $N_{healthy_a} = N_{healthy_{a-1}} - N_{healthy_{a-1}} * {}_{1}q_{a-1\_nhiv}$

Where $N_{hiv_a}, N_{syp_a}, N_{coinf_a}, N_{healthy_a}$ refer to the number of children with HIV only, congenital syphilis only, coinfection, and neither (i.e. "healthy") outcomes at 1 year, respectively, alive at age a.

The cumulative number of deaths by age $a \in \{0, \dots 19\}$ was calculated as follows:

(10.2.6) $D_a = D_{0_{syp}} + D_{a-1} + (N_{hiv_{a-1}} + N_{coinf_{a-1}}) * {}_{1}q_{a-1_{hiv}} + N_{nhiv_{a-1}} * {}_{1}q_{a-1\_nhiv}$

11 Infant DALYS

11. 1 Years lived with disability (YLDs)

11.1a YLDS for Infants with HIV infection only

(11.1.1) $YLDs_{hiv_a} =$

$$N_{hiv_a}\left(p_{ART} * p_{adh} * dw_{hiv_{tx}} + (1 - p_{ART} * p_{adh}) * dw_{hiv_{notx}} * pLE_{hiv} + dw_{AIDS_{notx}}(1 - pLE_{hiv_{notx}})\right)$$

Where $YLDs_{hiv_a}$ is the number of DALYs for infants with HIV infection only at time $a \in (1, \dots, 19)$, $dw_{hiv_{tx}}$ is the disability weight for HIV-infected individuals on ART, $dw_{hiv_{notx}}$ is the disability weight for HIV-infected individuals not on ART without AIDS, $dw_{AIDS_{notx}}$ is the disability weight for HIV-infected individuals not on ART with AIDS, and $pLE_{hiv_{notx}}$ is the proportion of the natural history of HIV prior to AIDS.

11.1b YLDS for infants with congenital syphilis only

Infants with congenital syphilis (clinical and asymptomatic) incur YLDs for congenital syphilis in the first 3 years only, calculated as:

(11.1.2) $YLDs_{syp_a} = I_3 * (N_{syp_a} * dw_{syp})$

Where I_3 is an indicator function for $a < 3$ and dw_{syp} is the disability weight for congenital syphilis.

11.1c YLDS for infants with syphillis-related low birth weight

Infants with excess low birth weight attributable to syphilis incur YLDs for low birth weight for the first year only, calculated as:

(11.1.3) $YLDs_{lbw_a} = I_1 * (N_{lbw} * dw_{lbw})$

Where I_1 is an indicator function for $a = 1$, N_{lbw} is the number of infants with low birth weight, and dw_{lbw} is the disability weight for low birth weight. Given the relatively low proportion of infants with low birth weight, we did not take steps to account for 'coinfection' of low birth and HIV.

11.1d YLDS for infants with congenital syphilis and HIV coinfection

Disability weights for coinfected individuals on ART, coinfected individuals not on ART without AIDS, and coinfected individuals not on ART with AIDS, were calculated as follows:

(11.1.4) $dw_{coinfHIV_{tx}} = 1 - \left(1 - dw_{HIV_{tx}}\right) * \left(1 - dw_{syp}\right)$

(11.1.5) $dw_{coinfHIV_{notx}} = 1 - (1 - dw_{HIV_{notx}}) * (1 - dw_{syp})$

(11.1.6) $dw_{coinf\,AIDS_{notx}} = 1 - (1 - dw_{AIDS_{notx}}) * (1 - dw_{syp})$

Infants with coinfection incur YLDs for coinfection for the first three years, followed by YLDs for HIV only, calculated as:

(11.1.7) $YLDs_{coinf_a} = N_{coinf_a} *$

$$\left(\begin{array}{c} I_3\left(p_{ART} * p_{adh} * dw_{coinf_{tx}} + \left(1 - p_{ART} * p_{adh}\right) * \left(dw_{coinfHIV_{notx}} * pLE_{hiv} + dw_{coinfAIDS_{notx}}\left(1 - pLE_{hiv_{notx}}\right)\right)\right) \\ + \\ (1 - I_3) * \left(p_{ART} * p_{adh} * dw_{hiv_{tx}} + \left(1 - p_{ART}\right) * \left(p_{adh} * dw_{hiv_{notx}} * pLE_{hiv} + dw_{AIDS_{notx}}\left(1 - pLE_{hiv_{notx}}\right)\right)\right) \end{array}\right)$$

Where I_3 is an indicator function for $a < 3$ and N_{coinf_a} is the number of infants with coinfection at time a. $dw_{coinfHIV_{tx}}$, $dw_{coinfHIV_{notx}}$, and $dw_{coinf\,AIDS_{notx}}$ are the disability weights for coinfected individuals on ART, coinfected individuals not on ART without AIDS, and coinfected individuals not on ART with AIDS, respectively.

11.1e Total YLDS

(11.1.8) $YLD_a = \sum YLD_{hiv_a} + YLD_{syp_a} + YLD_{lbw_a} + YLD_{coinf_a}$

11.2 Years of Life Lost (YLLs)

YLLs incurred at age $a \in \{0, \dots 19\}$ were calculated as:
(11.2.9) $YLL_a = D_a$

11.3 Total DALYS

Total DALYs were calculated by summing discounted YLDs and YLLs over all ages.
(11.3.10) $DALYs = \sum_{a=0}^{19} \frac{YLD_a + YLL_a}{(1+r)^{a+1}}$

Where r is the discount rate, 3%.

Appendix 3: Model Parameters

1 Maternal state transitions

1.1 Static across countries

Parameter	*Value*	*Source*
HIV RDT sensitivity, Ag-	0	Assumed
HIV RDT sensitivity, Ag+/Ab-	0	Assumed
HIV RDT sensitivity, Ab+	1	WHO PQ Public Report (2018)1
Dual RDT, HIV sensitivity, Ag-	0	Assumed
Dual RDT, HIV sensitivity, Ag+/Ab-	0	Assumed
Dual RDT, HIV sensitivity, Ab+	1	Van Den Heuvel (2019)
Duration of Ag- period (weeks)	2.3	Cohen (2010)[2]
Duration of Ag+/Ab- period (weeks)	0.7	Cohen (2010)[2]
Duration of early Ab+ period (higher viral load; weeks)	6	Assumed
Duration of acute maternal HIV infection (weeks)	9	Calculated[a]
Maternal PrEP use	0	Assumed
Proportion retained in ART at 1 year postpartum[b]	0.732	Haas (2016)[3]
Weekly risk of ART dropout[b]	0.0033	Calculated[c]
Proportional reduction in HIV incidence rate due to fully-adherent PrEP use	0.71	Heffron (2017)[4]
Gestational age in weeks, delivery	39	Assumed
Gestational age in weeks, early-postpartum	6	Assumed
Gestational age in weeks, mid-postpartum	14	Assumed
Gestational age in weeks, late-postpartum	26	Assumed
RPR sensitivity	0.8	Assumed
RPR specificity	0.98	WHO, 2013
TPHA sensitivity	1	Assumed
TPHA specificity	1	Assumed
Lab-based test sensitivity, syphilis	0.8	Calculated[d]
Lab-based test specificity, syphilis	1.0	Calculated[e]
Dual RDT, syphilis sensitivity	0.87	WHO PQ Public Report (2017)
Dual RDT, syphilis specificity	0.995	WHO PQ Public Report (2017)

Table A3a: [a]Sum of the Ag-, Ag+/Ab-, and early Ab+ durations. [b]Midpoint of 12 and 24 month retention. [c]Calculated assuming a constant proportion drops out each week. Product of RPR sensitivity and TPHA sensitivity. [e]RPR specificity + (1-RPR specifity)*TPHA specificity. Abbreviations: Ag-: antigen negative; Ag+: antigen positive; Ab-: antibody negative; Ab+: antibody positive; PrEP: maternal pre-exposure prophylaxis; ANC: antenatal care.

1.1 Country-specific

Parameter[a]	*Value*				*Source*			
	Kenya	*South Africa*	*Colombia*	*Ukraine*	*Kenya*	*South Africa*	*Colombia*	*Ukraine*
Weekly HIV incidence rate prior to first ANC visit	0.000331[b]	0.000227[b,i]	0.00001	0.000002[p]	Kinuthia (2015)[5]	Thomson (2018)[6], Kinuthia (2015)[5]	In-country source	UNAIDS Data (2018)[7,o]
Weekly HIV incidence rate after first ANC and up to delivery	0.000331[b]	0.000739[b,i]	0.00002	0.000004[p]	Kinuthia (2015)[5]	Thomson (2018)[6], Kinuthia (2015)[5]	Assumed	UNAIDS Data (2018)[7,o]
Weekly HIV incidence rate first 6 weeks postpartum	0	0	0	0	Assumed	Assumed	Assumed	Assumed
Weekly HIV incidence rate 6 weeks-12 months postpartum	0.00269[b]	0.0009[b]	0.000023	0.000003[q]	Kinuthia (2015)[5]	Thomson (2018)[6]	Assumed	UNAIDS Data (2018)[7,o]
Weekly syphillis incidence, duration of pregnancy	0.00008 [b]	0.00008 [b]	0.00008 [b]	0.000015 [b]	Newman (2015)	Newman (2015)	Newman (2015)	In-country source
Probability of attending first ANC visit	0.96[c]	0.94[c]	0.97	0.998	Kenya 2014 DHS[8], Sirengo (2014)[9]	South Africa 2016 DHS[10]	In-country source	Bozicevic (2018)[11]
Probability of attending second ANC visit	0.93[d]	0.78[k]	0.88	0.90	Kenya 2014 DHS[8]	South Africa 2016 DHS[10]	In-country source	Assumed
Probability of facility delivery	0.62	0.96	0.99	0.99	Kenya 2014 DHS[8]	South Africa 2016 DHS[10]	In-country source	Ukraine DHS (2007)[12]
Probability of HIV test kit stock-out	0.05	0.05	0	0	Assumed	Assumed	Assumed	Assumed
Probability of HIV test acceptance	0.84	0.98	0.89	0.97	Kohler (2014)[13]	Myer (2015)[14]	In-country source	Bozicevic (2018)[11]
Probability of receiving results of HIV test	0.98	0.98	1	1	Sirengo (2014)[15]	South Africa 2016 DHS[10]	Assumed	Assumed

Parameter[a]	*Value*				*Source*			
	Kenya	*South Africa*	*Colombia*	*Ukraine*	*Kenya*	*South Africa*	*Colombia*	*Ukraine*
Maternal ART use	0.91	0.87	0.88	0.95	UNAIDS Data (2019)[16]	UNAIDS Data (2019)[17]	PMTCT report[18]	UNAIDS Data (2019)[7]
Virally suppressed[g]	0.88	0.72[l]	0.88	0.88	Snippenburg (2017)[19]	Brittain (2019)[20]	Snippenburg (2017)[19]	Snippenburg (2017)[19]
Syphillis test coverage (inclusive of stock-outs)	0.73[r]	0.83	0.628	0.98	Assumption	Woldesenbet, 2017[21]	In-country source	In-country source
Weekly mortality rate for women of reproductive age, during pregnancy[h]	0.0001	0.0001	0.00002	0.00002	WHO life tables (2016)[22]	WHO life tables (2016)[22]	WHO life tables (2016)[22]	WHO life tables (2016)[22]
Weekly mortality rate, delivery through 6 weeks postpartum[i]	0.0006	0.0002	0.0001	0.00003	World Bank (2017)[23]	World Bank (2017)[23]	World Bank (2017)[23]	World Bank (2017)[23]
Weekly mortality rate, 6 weeks to 12 months postpartum[h]	0.0001	0.0001	0.00002	0.00002	WHO life tables (2016)[22]	WHO life tables (2016)[22]	WHO life tables (2016)[22]	WHO life tables (2016)[22]
Gestational age in weeks, first ANC	22	18[m]	15	10	McGrath (2018)[24]	South Africa 2016 DHS[10]	In-country source	Assumed
Gestational age in weeks, second ANC	33	36	24	28	Assumed	Assumed	Assumed	Assumed

Table A3b: [a]Incidence rates refer to incidence in the mother. [b]Calculated weekly incidence rate during prengancy from annual rate. [c]4% reported no ANC visits. [d]Calculated as 100 minus the percentage who had none/only one visit [e]Based on vaccine coverage, adjusted down by 2 percentage points to account for assumed vaccination delay. [f]Calculated as mid-point between 14 wk and 9 mo. [g]Among women on ART. [h]Average mortality rate of each age group between 15-49, divided by 52 to get weekly rate. [i]Calculated weekly rate from maternal mortality ratio. [j]Calculated as weighted average of 60% from Kinuthia (2015) and 40% from Thomson (2018). [k]Per South Africa 2016 DHS, 76% had 4 or more ANC visits and 2% had their first ANC in the 3rd trimester. [l]Percentage of women at 12 months postpartum who had VL ≥ 1000 copies/mL. [m]Calculated from median months pregnant at first ANC visit [n]Calculated from vaccination coverage. [o]Calculated from country-level HIV incidence. [p]Assumes 1/3 of incident infections during pregnancy occur before first ANC visit and 2/3 between first ANC visit and delivery. [q]Assumes half of incident infections during postpartum period occur between delivery and 6 months and half between 6 and 12 months. [r]Estimate from WHO syphillis estimation tool.

2 Infant outcomes

2.1 Static across countries

Parameter[a]	*Value*	*Source*
Incidence of infant HIV transmission *in utero*, established infection	0.0019[b]	Duri (2010)[25]
Incidence of infant HIV transmission *in utero*, recent infection	0.0165[c]	Marinda (2011)[26]
Incidence of infant HIV transmission, parturition and first six weeks postpartum, established infection, EBF	0.0226[d]	Zijenah (2004)[27]
Incidence of infant HIV transmission, parturition and first six weeks postpartum, recent infection, EBF	0.0294[e]	Assumed
Incidence of infant HIV transmission, mid post-partum, established infection, EBF	0.002[f]	Coovadia (2007)[28]
Incidence of infant HIV transmission, mid post-partum, recent infection, EBF	0.0054[g]	Liang (2009)[29]
Incidence of infant HIV transmission, late post-partum, established infection, EBF	0.0005	Assumed
Incidence of infant HIV transmission, late post-partum, recent infection, EBF	0.0054[g]	Liang (2009)[29]
Proportional reduction in HIV transmission due to infant ARV use	0.675	Kumwenda (2008)[30]
Proportional reduction in HIV transmission due to maternal viral load suppression	0.95 [h]	Kilewao (2009)[31]
Proportional reduction in HIV incidence rate of infant transmission due to NBF	1	Assumed
Probability of stillbirth, no syphillis	0.0003	Assumed
Probability of stillbirth, untreated or inadequately treated active maternal syphilis	0.21	Gomez (2012)[32]
Probability of neonatal death, untreated or inadequately treated active maternal syphilis	0.09	Gomez (2012)[32]
Probability of premature delivery /low birth weight, untreated or inadequately treated active maternal syphilis	0.06	Gomez (2012)[32]
Probability of clinical congenital syphilis case, untreated or inadequately treated active maternal syphilis	0.15	Gomez (2012)[32]
Reduction in stillbirth due to adequate treatment of active maternal syphilis	0.82	Blencowe (2011)[33]
Reduction in neonatal death due to adequate treatment of active maternal syphilis	0.80	Blencowe (2011)[33]
Reduction in premature delivery/low birth weight due to adequate treatment of active maternal syphilis	0.64	Blencowe (2011)[33]
Reduction in clinical congenital syphilis death due to adequate treatment of active maternal syphilis	0.97	Blencowe (2011)[33]
Probability of active infection, given positive test result	0.529[i]	Ham (2015)[34]
Adequate treatment cutoff, gestational weeks	32	Assumed
Disability weight, HIV on ART	0.078	Salomon (2015)[35]
Disability weight, HIV not on ART	0.274	Salomon (2015)[35]
Disability weight, AIDS	0.582	Salomon (2015)[35]
Disability weight, syphilis	0.315	Bristow (2016)[36]
Disability weight, low birth weight	0.106	Bristow (2016)[36]

Table A3c: [a]Incidence: weekly incidence rate. [b]Cumulative incidence of 7.46%, distributed over 40 weeks of pregnancy using decay formula and converted to rate; viral load suppression: undetectable viral load; NBF: complete breastfeeding avoidance; MF: mixed feeding. [c]Cumulative incidence of 14.15% distributed over 9 weeks of acute infection and converted to rate. [d]Cumulative incidence of 16% distributed over 7 weeks corresponding to delivery and first 6 weeks post-partum and converted to rate. [e]Assumed 30% higher than the established infection. [f]Six-month cumulative incidence among infants who were negative at 6 weeks (4%) distributed over 20 weeks (6 months-6 weeks) and converted to rate. [g]Cumulative incidence of 35.8% among women infected at delivery distributed over 66 weeks duration and converted to rate. [h]Estimate of infant transmission due to viral load supression is 4% at 6 months and 6% at 18 months so midpoint was used for transmission at 1 year. [i] Assumed midpoint between treponemal and non-treponemal tests. ***Abbreviations:*** EBF: Exclusive breastfeeding; early postpartum: first 6 weeks; mid postpartum: 6 weeks-6 months; late postpartum: 6-12 months; ART: antiretroviral treatment; ARV: infant antiretroviral prophylaxis**.**

2.2 Country-specific

Parameter	*Value* Kenya	*Value* South Africa	*Value* Colombia	*Value* Ukraine	*Source* Kenya	*Source* South Africa	*Source* Colombia	*Source* Ukraine
Probability infant receives ARVs	0.936[a]	0.99	0.96	0.98	McGrath (2018)[24]	In-country source	In-country source	Assumed
Probability HIV-infected infants receives ART[b]	0.61	0.63	0.579	0.95	UNAIDS (2019)[16]	UNAIDS (2019)[17]	Colombia PMTCT Report[18]	UNAIDS (2019)[7]
Probability of NBF early postpartum, HIV-	0.001	0.07[h]	0.031	0.046	In-country source	West (2019)[37]	UNICEF[38]	UNICEF[38]
Probability of NBF early postpartum, HIV+	0.025	0.34[h]	0.98	0.95	In-country source	West (2019)[37]	Colombia PMTCT Report[18]	Assumed
Probability of NBF mid postpartum, HIV-	0.0058[c]	0.19[h]	0.016	0.023	Assumed	West (2019)[37]	Assumed	Assumed
Probability of NBF mid postpartum, HIV+	0.21[c]	0.45[h]	0.98	0.99	Assumed	West (2019)[37]	Colombia PMTCT Report[18]	Assumed
Probability of NBF late postpartum, HIV-	0.009[d]	0.42[h]	0.0078	0.0115	In-country source	West (2019)[37]	Assumed	Assumed
Probability of NBF late postpartum, HIV+	0.33[d]	0.63[h]	0.98	0.99	In-country source	West (2019)[37]	Colombia PMTCT Report[18]	Assumed
Neonatal mortality, birth-6 weeks	0.0049[e]	0.0029[e]	0.0018[e]	0.001[e]	IHME (2017)[39]	IHME (2017)[39]	IHME (2017)[39]	IHME (2017)[39]
Infant mortality, >6 weeks-12 months	0.00031[f]	0.00025[f]	0.00009[f]	0.00006[f]	IHME (2017)[39]	IHME (2017)[39]	IHME (2017)[39]	IHME (2017)[39]
Survival to 1 year, HIV-	0.96[g]	0.97[g]	0.99[g]	0.99[g]	WHO life tables (2016)[22]	WHO life tables (2016)[22]	WHO life tables (2016)[22]	WHO life tables (2016)[22]
Survival to 1 year, HIV+ on ART	0.96[g]	0.97[g]	0.99[g]	0.99[g]	WHO life tables (2016)[22]	WHO life tables (2016)[22]	WHO life tables (2016)[22]	WHO life tables (2016)[22]
Survival to 1 year, HIV+ not on ART	0.65	0.65	0.65	0.65	Newell (2004)[40]	Newell (2004)[40]	Newell (2004)[40]	Newell (2004)[40]
Pediatric ART adherence	0.84	0.84	0.63	0.62	Kim (2014)[41]	Kim (2014)[41]	Kim (2014)[41]	Kim (2014)[41]
Adult ART coverage	0.69	0.62	0.74	0.52	UNAIDS (2019)[42]	UNAIDS (2019)[42]	UNAIDS (2019)[42]	UNAIDS (2019)[42]
Maternal syphillis treatment coverage	0.50	0.90	0.908	0.99	Assumed	Assumed	In-country source	In-country source

Table A3d:
[a]Calculated from probability of receiving ARVs conditional on HIV status, weighted by probabilities of HIV status (Table 3: 0.57*(13/188)+0.963*(175/188)). [b]Based on the percent of infants with early infant diagnosis. [c]Estimate derived by assuming a linear trend between measured breastfeeding practices at 6 weeks and 9 months. [d]Values measured at 9 months postpartum were assumed to be representative of the 6-12 month postpartum period. [e]Weighted average of early neonatal (0-6 days) and late neonatal (7-28 days) mortality rates, converted to daily rate, then converted to weekly probability. [f]Post-neonatal mortality rate converted to daily rate then weekly probability. [g]Assumed to be same as general population estimate. [h]Calculated as percentage not exclusively breastfeeding; assumes no mixed feeding. Abbreviations: NBF: no breastfeeding; early postpartum: first 6 weeks; mid postpartum: 6 weeks-6 months; late postpartum: 6-12 months; ART: antiretroviral treatment;

3 Maternal starting states

Parameter	*Value*				*Source*			
	Kenya	*South Africa*	*Colombia*	*Ukraine*	*Kenya*	*South Africa*	*Colombia*	*Ukraine*
Prevalence of HIV	0.061[a]	0.31	0.004	0.007	UNAIDS (2018) [16]	National sentinnel survey (2019) [43]	In-country source	Bozicevic (2018)[11]
Probability of HIV-infected pregnant women known HIV status	0.57[b]	0.608	0.67	0.98	Ronen (2017)[44]	National sentinnel survey (2019) [43]	Pranchniak-rincon (2016)[45]	Assumed
Prevalence of syphillis	1.2%[d]	2.0%	0.41%	1.2%	Assumed	In-country source	Newman (2015)[c]	Bozicevic (2018) [11]

Table A3e: [a]Dervied from percentage among women ages 15-49. [b]Calculated by weighted average of adolescents and adults. [c]Estimate for women, Region of the Americas.[d] From WHO syphillis estimation tool.

4 Costs

Parameter	*Value (2017 USD)*				*Source*			
	Kenya	*South Africa*	*Colombia*	*Ukraine*	*Kenya*	*South Africa*	*Colombia*	*Ukraine*
HIV Screening, 3rd generation rapid test [a]								
Labor[b]	1.92	7.15	3.70	0.39	Microcosting*	Microcosting* and in-country source	Microcosting* and in-country source	Microcosting* and in-country source
Test kit	0.52	0.40	2.88	3.50	Microcosting*	In-country source	In-country source	In-country source
Supplies	0.20	0.17	0.10[o]	0.10	Microcosting*	Microcosting* and in-country source	Microcosting* and assumption	Microcosting* and in-country source
Additional HIV screening costs, true-positive screening tests[a]								
Labor[c]	2.88	10.72	5.55	0.58	Microcosting*	Microcosting* and in-country source	Microcosting* and in-country source	Microcosting* and in-country source
Test kit (3rd gen)	0.60	0.50	2.88	3.50	Microcosting*	In-country source	In-country source	In-country source
Supplies	0.20	0.17	0.10[o]	0.10	Microcosting*	Microcosting* and in-country source	Microcosting* and assumption	Microcosting* and in-country source
Additional HIV screening costs, false-positive screening tests[a]								
Labor[d]	0.96	3.57	1.85	0.19	Microcosting*	Microcosting* and in-country source	Microcosting* and in-country source	Microcosting* and in-country source
Test kit (3rd gen + PCR)	25.23	30.43	72.88	19.50	Cintron (2017)[46]	In-country source	Assumption	In-country source
Supplies	0.20	0.17	0.10[o]	0.10	Microcosting*	Microcosting* and in-country source	Microcosting* and assumption	Microcosting* and in-country source
Syphilis screening, RPR screening per woman								
Labor[k, n]	2.18	7.74	4.01	0.42	Microcosting* and Kuznik (2013)[47]	In-country source and Kuznik (2013)[47]	In-country source and Kuznik (2013)[47]	In-country source and Kuznik (2013)[47]
Test kit	0.71	1.40	2.81[l]	0.11	Microcosting*	In-country source	Assumption	In-country source
Supplies	0.20	0.17	0.10[o]	0.10	Microcosting*	Microcosting* and in-country source	Microcosting* and assumption	Microcosting* and in-country source
Syphilis screening, TPHA (confirmatory)								

Parameter	*Value (2017 USD)*				*Source*			
	Kenya	*South Africa*	*Colombia*	*Ukraine*	*Kenya*	*South Africa*	*Colombia*	*Ukraine*
Labor[m, n]	0.35	1.19	0.62	0.07	Microcosting* and assumption	In-country source and assumption	In-country source and assumption	In-country source and assumption
Test kit	0.24	1.85	2.81[l]	1.07	Microcosting*	In-country source	Assumption	In-country source
HIV & Syphilis screening, Dual RDT								
Labor[b]	1.92	7.15	3.70	0.39	Microcosting*	Microcosting* and in-country source	Microcosting* and in-country source	Microcosting* and in-country source
Test kit	3.67	1.44	4.40	1.50	Microcosting*	In-country source	In-country source	In-country source
Supplies	0.20	0.17	0.10[o]	0.10	Microcosting*	Microcosting* and in-country source	Microcosting* and assumption	Microcosting* and in-country source
Per-week costs of maternal ART	4.86	4.79	18.89[h]	32.84	Larson (2018)[48]	Meyer-Rath (2019)[49]	In-country source	World Bank (2013)[50]
Total cost of a full course of infant ARVs	2.32[f]	3.82	52.10[i]	4.00	Bautista-Arrendondo (2013)[51]	Bautista-Arrendondo (2013)[51]	In-country source	In-country source
Per week costs of PrEP delivery	6.19[g]	6.19[g]	18.89[j]	19.38[j]	Roberts (2019)[52]	Roberts (2019)[52]	In-country source	Alistar (2014)[53]
Per week costs of infant ART, per infant	6.73	5.46	18.89[j]	32.84[j]	CDC (2013)[54]	Meyer-Rath (2019)	Assumed	Assumed
Benzyl benzathine penicillin injection, maternal treatment	0.64[p]	0.60	<0.01	3.00	Assumption	In-country source	In-country source	In-country source
Intravenous benzyl benzathine penicillin, infant treatment	1.42	1.42	1.42	1.42	MSH (2015)[55]	MSH (2015)[55]	MSH (2015)[55]	MSH (2015)[55]
Pediatric inpatient (per day)	8.41	8.41	8.41	8.41	WHO-CHOICE (2010)[56]	WHO-CHOICE (2010)[56]	WHO-CHOICE (2010)[56]	WHO-CHOICE (2010)[56]

Table A3f: [a]Cost applied at every visit where testing occurs. [b]Based on 30 minutes of nurse time for rapid test in Kenya from unpublished time and motion study (described in Appendix 4). [c]Based on 45 minutes of additional nurse time for true positives from unpublished time and motion study in Kenya. [d]Based on 15 minutes of additional nurse time for false positives from unpublished time and motion study in Kenya. [e]PCR costs are fully loaded. [f]Drug costs only. [g]Costs as implemented. [h]Cost for 9 months of pregnancy in pesos, converted to USD, then converted to weekly cost. [i]Cost for 42 days in pesos, divided by 6 to get weekly rate, then converted to USD. [j]Assumed to be the same as maternal ART costs. [k]Based on 17.5

minutes of nurse time and 15 minutes of lab worker time. [l]Assumed the cost was the same as a rapid test. [m]Assumed 5 additional minutes of lab technician time and no additional sample collection for TPHA. [n]Assumed annual salary for lab technician was equivalent to annual salary for nurse. [o]Assumed the same cost as Ukraine for gloves, cotton wool, and surgical spirit. [p]Assumed the same cost as South Africa *Microcosting details provided in Appendix 4. Abbreviations: ART: antiretroviral treatment; ARV: antiretroviral prophylaxis; PrEP: pre-exposure prophylaxis. HIV Testing assumptions: *Kenya*: third generation screening test = Alere Determine™ HIV-1/2, confirmatory test= First Response; tie-breaker test= viral load testing, defined in more detail in reference. *South Africa:* Confirmatory and tie-breaker testing consists of repeat of screening algorithm[57]. HIV Medication assumptions: *Kenya*: maternal ART= twice-daily zidovudine (AZT) + lamivudine (3TC) + lopinavir/ritonavir (LPV/r); infant ARV = 6 months nevirapine (NVP) and cotrimoxazole (CTX); infant ART= 3TC + AZT + NVP for infants <2 weeks old, abacavir (ABC) + 3TC + LPV/r for infants >2 weeks old; PrEP defined by reference in table. *South Africa:* maternal ART= tenofovir (TDF) + 3TC + dolutegravir (DTG); infant ARV= 6 weeks NVP; PrEP= TDF + emtricitabine (FTC); infant ART= ABC + 3TC + LPV/r. Exchange rate: World bank LCU per USD, converting June of year collected (if available) or reported (if collection year is unavailable) to June of 2017. Inflation adjustment: World bank GDP deflator.

References

1. WHO Prequalification of In Vitro Diagnostics Public Report. Product: Alere™ HIV Combo WHO reference number: PQDx 0243-013-00. September 2018, version 4.0. https://www.who.int/diagnostics_laboratory/evaluations/pq-list/hiv-rdts/180913_amended_final_pqpr_0033_013_00_v6.pdf?ua=1. Accessed.
2. Cohen MS, Gay CL, Busch MP, Hecht FM. The detection of acute HIV infection. *J Infect Dis.* 2010;202 Suppl 2:S270-277.
3. Haas AD, Tenthani L, Msukwa MT, et al. Retention in care during the first 3 years of antiretroviral therapy for women in Malawi's option B+ programme: an observational cohort study. *The lancet HIV.* 2016;3:e175-182.
4. Heffron R, McClelland RS, Balkus JE, et al. Efficacy of oral pre-exposure prophylaxis(PrEP) for HIV among women with abnormal vaginal microbiota: a post-hoc analysis of the randomized, placebo-controlled Partners PrEP Study. *Lancet HIV.* 2017;4:449-456.
5. Kinuthia J, Drake AL, Matemo D, et al. HIV acquisition during pregnancy and postpartum is associated with genital infections and partnership characteristics. *Aids.* 2015;29(15):2025-2033.
6. Thomson KA, Hughes J, Baeten JM, et al. Increased Risk of HIV Acquisition Among Women Throughout Pregnancy and During the Postpartum Period: A Prospective Per-Coital-Act Analysis Among Women With HIV-Infected Partners. *J Infect Dis.* 2018;218(1):16-25.
7. UNAIDS Country Factsheet. Ukraine. https://www.unaids.org/en/regionscountries/countries/ukraine. Published 2018. Accessed January 14, 2020.
8. *Kenya National Bureau of Statistics, Kenya Ministry of Health, Kenya National AIDS Control Council, Kenya Medical Research Institute, and Kenya National Council for Population and Development. Kenya Demographic and Health Survey 2014.* Rockville, MD, USA2015.
9. Sirengo M, Muthoni L, Kellogg TA, et al. Mother-to-child transmission of HIV in Kenya: results from a nationally representative study. *Journal of acquired immune deficiency syndromes (1999).* 2014;66 Suppl 1:S66-74.
10. *National Department of Health (NDoH), Statistics South Africa (Stats SA), South African Medical Research Council (SAMRC), and ICF. South Africa Demographic and Health Survey 2016: Key Indicators.* Pretoria, South Africa, and Rockville, Maryland, USA2017.
11. Bozicevic ID, Z. Report on Pre-Validation of Elimination of Mother-to-Child Transmission of HIV in Ukraine. December 2018. .
12. DHS Program. Ukraine Demographic and Health Survey. https://dhsprogram.com/publications/publication-FR210-DHS-Final-Reports.cfm. Published 2007. Accessed January 16, 2020, 2020.
13. Kohler PK, Okanda J, Kinuthia J, et al. Community-based evaluation of PMTCT uptake in Nyanza Province, Kenya. *PLoS One.* 2014;9(10):e110110.
14. Myer L, Phillips T, Manuelli V, McIntyre J, Bekker LG, Abrams EJ. Evolution of antiretroviral therapy services for HIV-infected pregnant women in Cape Town, South Africa. *Journal of Acquired Immune Deficiency Syndrome.* 2015.
15. Sirengo M, Muthoni L, Kellogg TA, et al. Mother-to-child transmission of HIV in Kenya: results from a nationally representative study. *Journal of Acquired Immune Deficiency Syndrome.* 2014;66 Suppl 1:S66-74.
16. UNAIDS Country Factsheets. Kenya. https://www.unaids.org/en/regionscountries/countries/kenya. Published 2018. Accessed January 27, 2020.
17. UNAIDS Country Factsheets. South Africa. https://www.unaids.org/en/regionscountries/countries/southafrica. Published 2018. Accessed January 27, 2020.
18. *ETMI - PLUS: Estrategia Nacional para la Eliminación de la Transmisión Materno Infantil del VIH, la sífilis congénita, la hepatitis B y la enfermedad de Chagas. Comportamiento de la Transmisión Materno Infantil del VIH en Colombia. Medición de la Cohorte 2017. Dirección de Promoción y Prevención Grupo de Sexualidad, Derechos Sexuales y Derechos Reproductivos. Colombia 29 de mayo de 2019*
19. Snippenburg W, Nellen F, Smit C, Wensing A, Godfried MH, Mudrikova T. Factors associated with time to achieve an undetectable HIV RNA viral load after start of antiretroviral treatment in HIV-1-infected pregnant women. *J Virus Erad.* 2017;3(1):34-39.
20. Brittain K, Mellins CA, Remien RH, et al. Impact of HIV-status disclosure on HIV viral load in pregnant and postpartum women on antiretroviral therapy. *JAIDS Journal of Acquired Immune Deficiency Syndromes.* 2019:1.
21. *WHO HIV/AIDS Programme. Antiretroviral drugs for treating pregnant women and preventing HIV infection in infants: towards universal access towards universal access. Recommendations for a public health approach.* Geneva, Switzerland2006.

22. World Health Organization. Country Life Tables. http://apps.who.int/gho/data/?theme=main&vid=60850. Published 2016. Accessed2019.
23. World Bank. Maternal mortality ratio modeled estimates, 2017. https://data.worldbank.org/indicator/sh.sta.mmrt. Published 2019. AccessedFebruary 28, 2019.
24. McGrath CJ, Singa B, Langat A, et al. Non-disclosure to male partners and incomplete PMTCT regimens associated with higher risk of mother-to-child HIV transmission: a national survey in Kenya. *AIDS Care.* 2018;30(6):765-773.
25. Duri K, Gumbo FZ, Kristiansen KI, et al. Antenatal HIV-1 RNA load and timing of mother to child transmission; a nested case-control study in a resource poor setting. *Virol J.* 2010;7:176.
26. Marinda ET, Moulton LH, Humphrey JH, et al. In utero and intra-partum HIV-1 transmission and acute HIV-1 infection during pregnancy: using the BED capture enzyme-immunoassay as a surrogate marker for acute infection. *Int J Epidemiol.* 2011;40(4):945-954.
27. Zijenah LS, Moulton LH, Iliff P, et al. Timing of mother-to-child transmission of HIV-1 and infant mortality in the first 6 months of life in Harare, Zimbabwe. *Aids.* 2004;18(2):273-280.
28. Coovadia HM, Rollins NC, Bland RM, et al. Mother-to-child transmission of HIV-1 infection during exclusive breastfeeding in the first 6 months of life: an intervention cohort study. *Lancet.* 2007;369(9567):1107-1116.
29. Liang K, Gui X, Zhang YZ, Zhuang K, Meyers K, Ho DD. A case series of 104 women infected with HIV-1 via blood transfusion postnatally: high rate of HIV-1 transmission to infants through breast-feeding. *J Infect Dis.* 2009;200(5):682-686.
30. Kumwenda NI, Hoover DR, Mofenson LM, et al. Extended antiretroviral prophylaxis to reduce breast-milk HIV-1 transmission. *N Engl J Med.* 2008;359(2):119-129.
31. Kilewo C, Karlsson K, Ngarina M, et al. Prevention of mother-to-child transmission of HIV-1 through breastfeeding by treating mothers with triple antiretroviral therapy in Dar es Salaam, Tanzania: the Mitra Plus study. *J Acquir Immune Defic Syndr.* 2009;52(3):406-416.
32. Gomez GB, Kamb ML, Newman LM, Mark J, Broutet N, Hawkes SJ. Untreated maternal syphilis and adverse outcomes of pregnancy: a systematic review and meta-analysis. *Bulletin of the World Health Organization.* 2013;91:217-226.
33. Blencowe H, Cousens S, Kamb M, Berman S, Lawn JE. Lives Saved Tool supplement detection and treatment of syphilis in pregnancy to reduce syphilis related stillbirths and neonatal mortality. *BMC public health.* 2011;11(S3):S9.
34. Ham DC, Lin C, Newman L, Wijesooriya NS, Kamb M. Improving global estimates of syphilis in pregnancy by diagnostic test type: A systematic review and meta-analysis. *International Journal of Gynecology & Obstetrics.* 2015;130:S10-S14.
35. Salomon JA, Haagsma JA, Davis A, et al. Disability weights for the Global Burden of Disease 2013 study. *Lancet Glob Health.* 2015;3(11):e712-723.
36. Bristow CC, Larson E, Anderson LJ, Klausner JD. Cost-effectiveness of HIV and syphilis antenatal screening: a modelling study. *Sex Transm Infect.* 2016;92(5):340-346.
37. West NS, Schwartz SR, Yende N, et al. Infant feeding by South African mothers living with HIV: implications for future training of health care workers and the need for consistent counseling. *Int Breastfeed J.* 2019;14:11.
38. UNICEF. Breastfeding A mother's Gift for Every Child. Published 2018. Accessed January 16, 2020.
39. Institute for Health Metrics and Evaluation. Global Burden of Disease Estimates. http://ghdx.healthdata.org/gbd-results-tool. Published 2017. Accessed2019.
40. Newell ML, Coovadia H, Cortina-Borja M, et al. Mortality of infected and uninfected infants born to HIV-infected mothers in Africa: a pooled analysis. *Lancet.* 2004;364(9441):1236-1243.
41. Kim SH, Gerver SM, Fidler S, Ward H. Adherence to antiretroviral therapy in adolescents living with HIV: systematic review and meta-analysis. *AIDS.* 2014;28(13):1945-1956.
42. UNAIDS AIDSinfo. https://aidsinfo.unaids.org/. Published 2019. Accessed March 19, 2020.
43. Woldesenbet SA, Kufa T, Lombard C, et al. The 2017 National Antenatal Sentinel HIV Survey, South Africa, National Department of Health. 2019.
44. Ronen K, McGrath CJ, Langat AC, et al. Gaps in Adolescent Engagement in Antenatal Care and Prevention of Mother-to-Child HIV Transmission Services in Kenya. *J Acquir Immune Defic Syndr.* 2017;74(1):30-37.
45. Prachniak-Rincon C, Villar de Onis J. HIV and the Right to Health in Colombia. *Health Hum Rights.* 2016;18(2):157-169.
46. Cintron C, Mudhune V, Haider R, et al. *Costs of HIV Viral Load and Early Infant Diagnosis Testing in Kenya.* Health, Finance & Governence; USAID;2017.

47. Kuznik A, Lamorde M, Nyabigambo A, Manabe YC. Antenatal syphilis screening using point-of-care testing in Sub-Saharan African countries: a cost-effectiveness analysis. *PLoS Med.* 2013;10(11):e1001545.
48. Larson BA, Bii M, Halim N, Rohr JK, Sugut W, Sawe F. Incremental treatment costs for HIV-infected women initiating antiretroviral therapy during pregnancy: A 24-month micro-costing cohort study for a maternal and child health clinic in Kenya. *PloS one.* 2018;13(8):e0200199.
49. Meyer-Rath G, van Rensburg C, Chiu C, Leuner R, Jamieson L, Cohen S. The per-patient costs of HIV services in South Africa: Systematic review and application in the South African HIV Investment Case. *Plos One.* 2019;14(2):e0210497.
50. The World Bank. *Ukraine HIV Program Efficiency Study: Can Ukraine improve value for money in HIV service delivery?* Washington, DC2013.
51. Bautista-Arredondo S, Sosa-Rubí SG, Opuni M, et al. Costs along the service cascades for HIV testing and counselling and prevention of mother-to-child transmission. *AIDS.* 2013;30(16):2495-2504.
52. Roberts DA, Barnabas RV, Abuna F, et al. The role of costing in the introduction and scale-up of HIV pre-exposure prophylaxis: evidence from integrating PrEP into routine maternal and child health and family planning clinics in western Kenya. *J Int AIDS Soc.* 2019;22 Suppl 4:e25296.
53. Alistar SS, Owens DK, Brandeau ML. Effectiveness and cost effectiveness of oral pre-exposure prophylaxis in a portfolio of prevention programs for injection drug users in mixed HIV epidemics. *PloS one.* 2014;9(1):e86584.
54. CDC, Kenya Ministry of Health. *The Cost of Comprehensive HIV Treatment in Kenya.* 2013.
55. *MSH (Management Science for Health). 2015. International Durg Price Indicator Guide, 2014 Edition. MEdford, Mass,: MSH.*
56. *WHO-CHOICE estimats of cost for inpatient and outpatient health service delivery.*
57. *South Africa National Department of Health. National HIV Testing Services: Policy.* 2016.

Appendix 4: Microcosting methods

We collected repeat cost data were between June and November 2017 in Kenya, from the provider perspective. This costing analysis was nested in an ongoing study implementing repeat maternal testing during HIV and pregnancy using a 4th generation screening test.

We conducted time and motion studies to micro-cost the cost of personnel, and we abstracted the costs of HIV test kits and other supplies (e.g. ,surgical fluid) from clinic registers at Ahero County and Bondo sub-County Hospitals in western Kenya. We removed all research-related costs were removed from our cost estimates
